# Perspective Exploring Novel Associations of IL-18 Levels as a Mediator of the Causal Links between Major Depression and Reproductive Health

**DOI:** 10.1155/2024/9234876

**Published:** 2024-08-05

**Authors:** Mengying Li, Kaibo Sun, Yunyun Mei, Keyan Liu, Lei Chen, Yihong Guo

**Affiliations:** ^1^Center of Reproductive Medicine, The First Affiliated Hospital of Zhengzhou University, Zhengzhou, China; ^2^Department of Orthopedics Surgery, West China Hospital of Sichuan University, Chengdu, Sichuan, China; ^3^Fudan University Shanghai Cancer Center (Xiamen Hospital), Xiamen, China; ^4^Health Management Center, The First Affiliated Hospital of Zhengzhou University, Zhengzhou, China

## Abstract

This research has suggested a link between major depressive disorder (MDD) and infertility, with interleukin-18 (IL-18) being proposed as a potential mediator due to its connections to both conditions. A Mendelian randomization (MR) approach was utilized in this study, which drew on genetic data from 500,199 European participants studied for MDD, along with additional IL-18 and reproductive health data from the FinnGen consortium and GWAS datasets. Single nucleotide polymorphisms were employed as instrumental variables to examine the causal relationships between MDD, genetically predicted IL-18 levels, and infertility. In our study, bidirectional MR analysis revealed a significant inverse causal relationship between MDD and genetically predicted IL-18 levels, with a higher genetic predisposition to MDD, correlating with reduced IL-18 levels (*β*: −0.40; 95% confidence interval (CI): −0.69 to −0.11; *P* = 7.09 × 10^−3^). Additionally, MDD is found to significantly increase the risk of female infertility. Notably, genetically predicted IL-18 levels demonstrated a protective effect against female infertility (odds ratio (OR): 0.92; 95% CI: 0.86–0.98; *P* = 1.17 × 10^−2^). Mediation analysis indicated that genetically predicted IL-18 levels partially mediated the impact of MDD on female infertility associated with cervical, vaginal, other or unspecified origin, accounting for up to 14.61% of this effect. No evidence of pleiotropy or heterogeneity was detected. The role of genetic predispositions to MDD in influencing genetically predicted IL-18 levels, and subsequently, female infertility, was highlighted by our study, offering insights into the complex interplay between mental health and reproductive biology. These findings contribute to a deeper understanding of the genetic and molecular pathways influencing these conditions, suggesting new directions for research and potential therapeutic interventions.

## 1. Introduction

Infertility affects a significant portion of the population, with varying prevalence across the globe [1, 2]. Female infertility is a complex condition with various contributing factors. Biological elements, such as hormonal imbalances and reproductive health disorders, are well-established contributors [3]. Additionally, there is growing interest in the potential influence of psychological aspects, including stress and mental health conditions like major depressive disorder (MDD) [4, 5, 6]. MDD and anxiety can profoundly impact hormonal balance and immune function by interleukins, potentially leading to conditions such as polycystic ovary syndrome (PCOS), autoimmune disorders, and endometriosis [7], which are directly linked to fertility issues [8, 9, 10]. While there is some evidence suggesting a correlation between these psychological factors and fertility outcomes, it is important to note that current research does not conclusively establish causality [11, 12].

While the connection between MDD and female infertility has been suggested [13], the precise mechanisms underlying this intricate relationship remain incompletely understood. MDD is often characterized by a mild inflammatory signature, as evidenced by increased plasma concentrations of proinflammatory cytokines, including interleukin-18 (IL-18) [14, 15]. Elevated levels of IL-18 have been linked to an intensification of inflammatory responses, particularly in inducing interferon-gamma (IFN-*γ*) production, which is a notable feature of depressive states [16, 17]. This exacerbation of inflammation can lead to various physiological changes associated with MDD, such as altered neurotransmitter metabolism and disrupted neuroendocrine function [18, 19]. Furthermore, abnormal levels of IL-18 in reproductive tissues can disrupt the delicate balance of cytokines required for successful implantation and pregnancy [20]. Elevated IL-18 levels have been associated with conditions like endometriosis and PCOS, both of which are linked to infertility [21, 22]. The involvement of IL-18 may be through its impact on hormonal regulation, particularly in the hypothalamic–pituitary–gonadal axis, or influence immune mechanisms through natural killer cells and autoantibodies, which are both crucial for reproductive health [23, 24, 25].

The mediating role of IL-18 lies in its potential to act as a biological bridge between MDD and female infertility. The significance of IL-18 extends to pregnancy, where it contributes to a Th1–Th2 cooperation phenomenon, indicative of a predominantly Th2-type lymphocyte response [26]. This cytokine is also crucial in the “decidualization” process at the implantation site, suggesting its importance in early pregnancy stages [27, 28]. Alterations in IL-18 levels may be a primary event in the pathogenesis of immunological infertility, influencing recurrent spontaneous abortion and the efficacy of autoantibodies [29].

Understanding how IL-18 influences this relationship may provide valuable insights into the underlying mechanisms and offer new avenues for addressing female infertility in individuals affected by MDD. Further complicating this landscape is the bidirectional relationship between infertility and MDD. Infertility can exacerbate symptoms of depression and anxiety, creating a vicious cycle where psychological distress further aggravates fertility issues. This underscores the necessity for a holistic treatment approach that addresses both mental, and reproductive health. Enlightening the associations of IL-18 Levels as a mediator of the causal links between major depression, anxiety and reproductive health could potentially have an effect on future clinical practice by introducing magnesium supplementation for improving women's overall and reproductive health and well-being [30].

To elucidate the causal relationships and underlying mechanisms, this paper employs Mendelian randomization (MR) analysis, using genetic variants as proxies for exposure. MR allows for a robust inference of causality, avoiding confounders typical in observational studies [31, 32]. The principle of MR relies on the assumption that genetic variants, which are randomly allocated at conception, are not influenced by environmental factors or disease states, providing a form of natural randomization [33, 34]. The study utilizes both two-sample and multivariable MR to isolate specific effects of MDD and IL-18 on infertility, controlling for pleiotropic influences where genetic variants affect multiple traits. Through mediating MR, we aim to quantify the proportion of MDD's effect on infertility mediated through IL-18, shedding light on intermediary mechanisms.

## 2. Materials and Methods

### 2.1. Theoretical Framework

**Figure 1:**
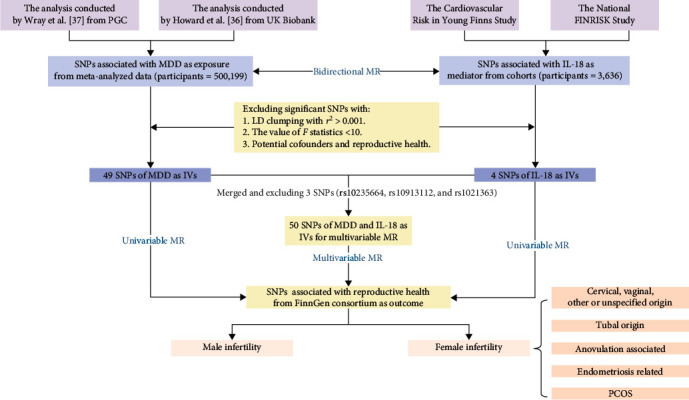
The detailed process of the study. PGC, psychiatric genomics consortium; MDD, major depressive disorder; SNP, single nucleotide polymorphisms; LD, linkage disequilibrium; IL-18, interleukin-18; IVs, instrumental variables; MR, Mendelian randomization; PCOS, polycystic ovary syndrome.

**Figure 2:**
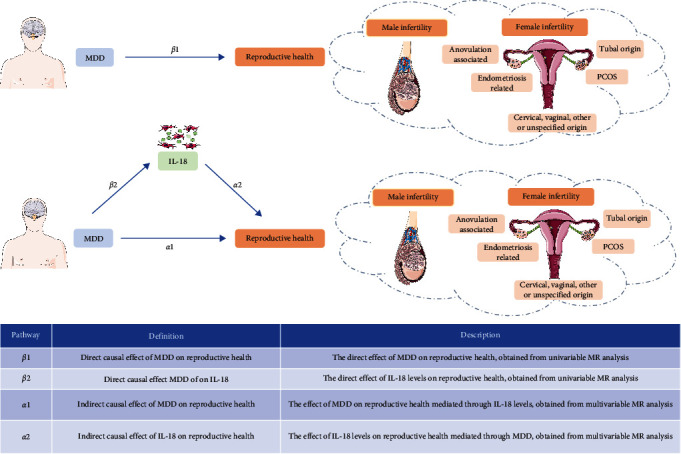
The framework of MR analysis in the study. Reproductive health includes both male and female infertility. Female infertility is categorized into subtypes such as cervical, vaginal, other or unspecified origin, tubal origin, anovulation-associated, endometriosis-related, and PCOS. MDD, major depression disorder; IL-18, interleukin-18; PCOS, polycystic ovary syndrome.

This study employs an MR design and is categorized as an observational study. The detailed process of the study is shown in Figure 1. The basic information on the genome-wide association studies (GWAS) of exposure and outcome applied in this study is presented in *Supplementary table 1*. Initially, we employed a bidirectional MR approach to investigate the causal relationships between MDD and IL-18 levels. Subsequently, separate univariable MR analyses were conducted to assess the direct effect of MDD on reproductive health (*β*1). Additionally, we evaluated the direct effect of MDD on IL-18 levels (*β*2). To comprehensively evaluate the mediation effect, multivariable MR analyses for MDD and IL-18 levels were performed separately. The indirect causal effect of MDD on reproductive health, adjusted for IL-18 levels, was represented as *α*1. Similarly, the indirect causal effect of IL-18 levels on reproductive health, adjusted for MDD, was indicated by *α*2. The mediating effect of IL-18 between MDD and reproductive health was *α*2 × *β*2. The proportion of MDD on reproductive health mediated by IL-18 levels was calculated as (*α*2 × *β*2)/*β*1. This complex analysis framework is visually represented in Figure 2.

### 2.2. MDD and IL-18 as Exposure

The selected single nucleotide polymorphisms (SNPs) for MDD are derived from the most recent and extensive GWAS available (reference number: ieu-b-102) [35]. The total number of individuals in these data is 500,199 (170,756 cases and 329,443 controls), including Howard et al.'s [36] analysis of UK Biobank data (127,552 cases and 233,763 control) and the meta-analysis of Wray et al. [37] from Psychiatric Genomics Consortium (PGC) (43,204 cases and 95,680 controls). All participants in these studies are of European ancestry, ensuring population homogeneity and reducing potential confounding due to population stratification.

The relevant summary statistics data on IL-18 levels come from the GWAS, which combined genetic variants associated with cytokine levels from two cohorts [38]: the Cardiovascular Risk in Young Finns Study and FINRISK 2002, with 3,636 individuals of European descent. The Cardiovascular Risk in Young Finns Study is one of the largest follow-up studies tracking cardiovascular risk from childhood to adulthood, and FINRISK 2002 is part of the broader FINRISK cohorts, assessing risk factors for various chronic diseases (e.g., diabetes and cancer). The instrumental variables (IVs) for IL-18 is representative of general population data. More information on this GWAS is available at https://gwas.mrcieu.ac.uk/datasets/ebi-a-GCST004441/.

Genetic variants associated with MDD and IL-18 levels serve as IVs. We initially selected SNPs associated with IVs, all of which demonstrated genome-wide significance (*P* < 5 × 10^−8^). To mitigate the effects of strong linkage disequilibrium (LD), we applied a stringent LD threshold (*r*^2^ < 0.001). The strength of these instruments was evaluated using *F*-statistics, excluding any SNP with an *F* < 10 due to weak instrument strength. For SNPs not present in the GWAS dataset, the LDlink platform was utilized to identify suitable proxy SNPs, of which the commonly used cutoff for LD *r*^2^ is 0.8, ensuring comprehensive and robust genetic instruments for this analysis. When calculating the direct effect of MDD on reproductive health using MR, we selected SNPs that are only associated with MDD and not with the mediator IL-18 levels [39]. Additionally, we carefully excluded SNPs that are specifically related to outcomes like PCOS and endometriosis and potential confounding factors such as thyroid dysfunction; we utilized the PhenoScanner database (http://www.phenoscanner.medschl.cam.ac.uk/), a comprehensive resource for exploring the associations between genetic variants and phenotypes. Finally, 49 SNPs for MDD and 4 SNPs for IL-18 levels collected as IVs were selected for MR analyses. Detailed information on the IVs is shown in *Supplementary table 2*.

### 2.3. Reproductive Health as Outcomes

We leverage the ninth release of the FinnGen consortium, a comprehensive dataset combining Finnish genetic data with digital health records [40]. This study did not broadly encompass reproductive health in general but focused on the aspect of infertility. Genetic variations associated with outcomes encompass both male and female infertility. Female infertility encompasses various subcategories, including cervical, vaginal, other or unspecified origin, tubal origin, anovulation-associated, endometriosis-related, and PCOS. Detailed participant information, genotyping methods, sample sizes, and definitions for female and male infertility types are available on the FinnGen website (https://www.finngen.fi/).

### 2.4. Statistical Analysis

To enhance the robustness of our findings, we employed the inverse variance-weighted (IVW) method as the primary approach, MR-Egger and weighted median methods in the univariable MR. Additionally, we conducted a multivariable MR analysis, including the multivariable IVW, MR-Egger, and weighted median and MR-Lasso methods, to further enhance the validity and reliability of our results.

Sensitivity analyses were conducted to ensure the validity and robustness of our results. Heterogeneity among the SNPs was assessed using Cochrane's *Q* statistic [41], with significance indicated by a *P* value < 0.05. A leave-one-SNP-out analysis was performed to detect potential outlier SNPs influencing causal associations [42]. The Egger-intercept test was employed to examine pleiotropy, while funnel plots visually assessed its presence [43].

To address multiple testing, we applied the Bonferroni correction since eight outcomes were considered [44]. Significance was defined as associations with *P* < 7.14 × 10^−3^ (0.05/7), with *P* values between 0.05 and 7.14 × 10^−3^ considered suggestive causal associations. We utilized R version 4.0.5 for data formatting and conducted all analyses using the “TwoSampleMR” and “MVMR” packages in R (version 4.1.3; Lucent Technologies; New Jersey, USA).

## 3. Results

### 3.1. Causal Effect between MDD and IL-18

We performed a bidirectional MR analysis between MDD on IL-18 levels. Using the IVW method, the analysis identified a significant negative causal effect of MDD on IL-18 levels, with genetically higher MDD risk associated with lower IL-18 levels (*β*: −0.40; 95% confidence interval (CI): −0.69 to −0.11; *P* = 7.09 × 10^−3^). In contrast, no causal effect of IL-18 levels on MDD (odds ratio (OR): 1.00; 95% CI: 0.97–1.02; *P*=0.84) was detected, indicating a one-way association. The detailed results of the bidirectional MR analysis and sensitivity analyses are shown in Table 1.

### 3.2. Causal Effect of MDD on Infertility

**Figure 3:**
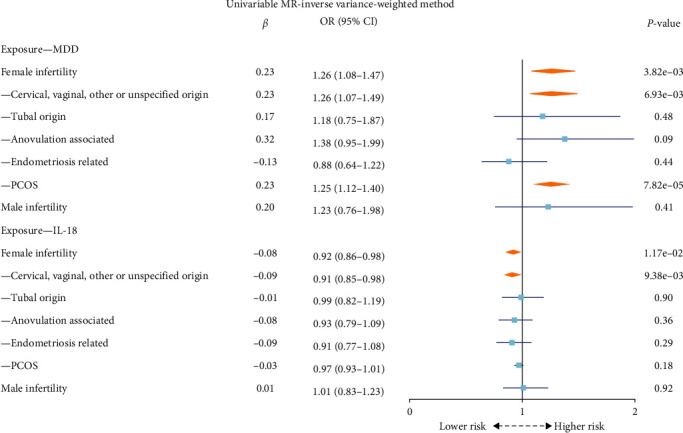
The forest plot of the causal effect of MDD and IL-18 on infertility using univariable MR inverse variance-weighted method. MDD, major depressive disorder; IL-18, interleukin-18; MR, Mendelian randomization; PCOS, polycystic ovary syndrome.

In examining the causal influence of MDD on infertility in univariable IVW MR analysis, as shown in Figure 3, a significant positive effect was observed on female infertility (OR: 1.26; 95% CI: 1.08–1.47; *P* = 3.82 × 10^−3^), with further analysis showing notable effects on specific subtypes, including cervical, vaginal, other or unspecified origin (OR: 1.26; 95% CI: 1.07–1.49; *P* = 6.93 × 10^−3^), and PCOS (OR: 1.25; 95% CI: 1.12–1.40; *P* = 7.82 × 10^−5^). No such effects were found for other subtypes. There was also no significant causal effect between MDD and male infertility.

**Figure 4:**
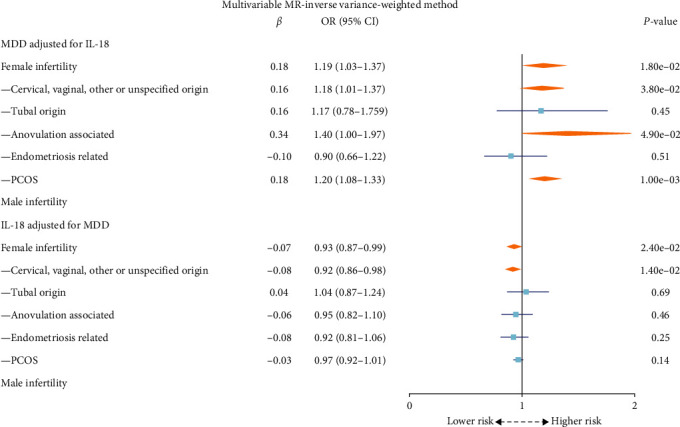
The forest plot of the causal effect of MDD and IL-18 on infertility using multivariable MR inverse variance-weighted method. MDD, major depressive disorder; IL-18, interleukin-18; MR, Mendelian randomization; PCOS, polycystic ovary syndrome.

Adjusting for IL-18 in the multivariable IVW MR analysis in Figure 4, MDD still exhibited a significant effect on female infertility (OR: 1.19; 95% CI: 1.03–1.38; *P* = 1.80 × 10^−2^), with subgroup analyses supporting this causal relationship for infertility linked to cervical, vaginal, other or unspecified origin (OR: 1.18; 95% CI: 1.01–1.38; *P* = 3.80 × 10^−2^) and PCOS-linked infertility (OR: 1.20; 95% CI: 1.08–1.33; *P* = 1.00 × 10^−3^).

### 3.3. Causal Effect of IL-18 Levels on Infertility

Using univariable IVW MR analysis in Figure 3, IL-18 levels showed a protective association with female infertility risk (OR: 0.92; 95% CI: 0.86–0.98; *P* = 1.17 × 10^−2^), which remained consistent after adjusting for MDD in multivariable IVW MR analysis (OR: 0.93; 95% CI: 0.87–0.99; *P* = 2.40 × 10^−2^) in Figure 4. Subsequent analyses were undertaken to dissect the causal relationship by infertility subtype. Specifically, IL-18 levels exhibited a statistically suggestive causal effect on female infertility associated with cervical, vaginal, other or unspecified origin (OR: 0.91; 95% CI: 0.85–0.98; *P* = 9.38 × 10^−3^), as observed using the multivariable IVW method (OR: 0.92; 95% CI: 0.86–0.98; *P* = 1.40 × 10^−2^). No significant causal relationship was found between IL-18 and male infertility.

The detailed results of the other univariable and multivariable analyses examining the relationship between MDD and IL-18 on reproductive outcomes are presented in *Supplementary table 3* and *Supplementary table 4*. Additionally, scatter plots illustrating univariable analysis are available in *Supplementary figure 1, Supplementary figure 2, Supplementary figure 3*, and *Supplementary figure 4*.

### 3.4. The Mediating Role of IL-18 in the MDD-Infertility Link

Regarding the mediating role of IL-18 in the link between MDD and infertility, our initial findings suggested a partial mediation effect. The mediation effect size was 2.92 × 10^−2^, explaining ~12.70% of the risk of infertility attributed to MDD.

Further exploration of different types of female infertility revealed that IL-18 partially mediated the effect of MDD on female infertility associated with cervical, vaginal, other or unspecified origin. The mediation effect size in these cases was 3.36 × 10^−2^, indicating that IL-18 acts as an intermediary in ~14.61% of this relationship.

### 3.5. Sensitivity Analyses in the MR Analysis

The robustness of our findings is further cemented by rigorous sensitivity analyses that showed no evidence of heterogeneity or horizontal pleiotropy. These analyses reinforce the validity of our results, suggesting that the genetic instruments used in our MR analyses are appropriate and that our causal estimates are reliable. Additional details, including MR-Egger intercept and Cochran's *Q* tests, are available in *Supplementary table 5* and *Supplementary table 6*. Furthermore, funnel plots used for heterogeneity testing are provided in *Supplementary figure 5, Supplementary figure 6, Supplementary figure 7*, and *Supplementary figure 8*. The results of the leave-one-SNP-out analysis are shown in *Supplementary figure 9*, *Supplementary figure 10, Supplementary figure 11*, and *Supplementary figure 12*.

## 4. Discussion

### 4.1. Principal Findings

The most important finding in our study is the role of IL-18 as a partial mediator in this association, contributing to ~12.70% of the infertility risk attributable to MDD. Notably, IL-18's mediation was particularly pronounced in infertility cases with cervical, vaginal, and other or unspecified origins, accounting for ~14.61% of the MDD-infertility link.

### 4.2. Results in the Context of What Is Known

Our univariable MR analysis revealed a significant positive causal relationship between MDD and female infertility, particularly in cervical, vaginal, and other or unspecified origins, as well as in cases of PCOS. These subtype-specific insights underscore the multifaceted nature of MDD's impact on female reproductive health. The observed elevated prevalence of mental health and metabolic disorders among women with PCOS, as consistently reported in a previous study [45], further emphasizes the significance of the association between PCOS and mental health disorders. This connection has been linked to detrimental effects on self-care practices and the adoption of unhealthy lifestyle behaviors.

Several studies have found a significant increase in IL-18 levels in individuals with MDD compared to the control group [46, 47, 48]. However, in our article, we found that individuals with a higher genetic predisposition to MDD exhibited reduced IL-18 levels. Interestingly, we did not observe a reverse causal influence, indicating a unidirectional impact of MDD on IL-18 levels. Biological mechanisms could influence the relationship between MDD and IL-18 levels, with the timing and chronicity of the depression playing a critical role. Acute episodes of MDD might show elevated inflammatory markers, including IL-18, due to stress and immune activation [49]. In contrast, chronic or long-term depression could lead to different immune responses, potentially resulting in lower IL-18 levels due to immune system exhaustion or adaptation [50]. This finding supports the existing understanding of the close interaction between mental distress and immune function via the release of cytokines such as IL-18, which regulate immune responses and inflammation, as previous studies have presented [16, 51].

### 4.3. Clinical Implications

Successful implantation of an embryo relies on the precise activation of the endometrial immune system, which plays a crucial role in facilitating embryo acceptance and implantation [52, 53]. Interleukins, key components of the uterine microenvironment, are involved in mediating communication between the embryo and the maternal immune system [54, 55, 56]. They influence important processes such as decidua formation, embryo acceptance, trophoblast invasion, and placental development [57, 58, 59]. The production of ILs in the endometrium is essential for creating an optimal environment for implantation. However, aberrant IL production can disrupt this delicate balance and hinder the implantation process, even with high-quality embryos, leading to recurrent implantation failure [60, 61, 62]. The imbalanced production of ILs and the resulting elevation of proinflammatory cytokines in the endometrium mimic the immune response seen in alloimmune graft rejection. This immune imbalance ultimately leads to embryo rejection and subsequent pregnancy failure [63]. The involvement of the inflammasome complex, particularly the nucleotide binding and oligomerization domain-like receptor (NLR) family, in the immunopathogenesis of PCOS is noteworthy. Studies have shown a significant increase in the expression of NALP3 in PCOS, which is strongly correlated with IL-18 levels. The activation of the NLR family apoptosis inhibitory protein (NAIP) inflammasome is likely responsible for the elevated expression of IL-18 and IL-1*β*, indicating a potential critical role of the inflammasome in the immunopathology of PCOS [64]. Similarly, elevated NLR family pyrin domain-containing 3 (NALP3) inflammasome levels have been observed in the ovaries of endometriosis patients. This increase in NLRP3 inflammasome activity is associated with significantly higher IL-18 levels, which may affect female fertility [21]. Unlike previous studies that have shown elevated IL-18 levels in patients associated with female infertility conditions like PCOS, our MR study found high levels of IL-18 appeared to be protective against infertility. One possible explanation for this discrepancy is that elevated IL-18 in certain conditions, such as PCOS, might represent a compensatory or protective mechanism. In this context, the elevation of IL-18 could be a response to underlying inflammation and metabolic disturbances, aiming to mitigate further damage or dysfunction.

Notably, IL-18 levels were only found to be altered in female infertility, suggesting the influence of sex-specific factors. This finding aligns with previous research indicating sex differences in immune function and stress responses [65, 66]. These sex-specific factors contribute to the observed disparities in the association between immune system dysregulation and IL-18 levels across sexes.

Modern medical infertility treatment options include ovulation-inducing hormones and in vitro fertilization (IVF) [67]. However, these treatments can be associated with adverse events such as multiple pregnancies, ovarian hyperstimulation syndrome, and ovarian cancer. Moreover, if the success rate of IVF is low due to age or repeated unsuccessful attempts, there are limited alternatives in modern medicine.

### 4.4. Research Implications

Our study has important implications for clinical practice. Screening for and managing depression in women seeking fertility treatments is crucial, given the significant association between MDD and female infertility. Furthermore, considering the mediating role of IL-18, exploring anti-inflammatory interventions as adjunctive treatments for MDD-related infertility could be beneficial. However, it is important to mention that the exact mechanisms by which IL-18 influences infertility are not fully understood. Therefore, further research is necessary to address the complex interplay of psychological and immunological factors in female infertility and potentially guide the exploration of therapeutic implications.

### 4.5. Strengths and Limitations

Our study is the first MR investigation exploring IL-18 as a partial mediator in the association between MDD and female infertility. We provided subtype-specific insights, particularly in cases with cervical, vaginal, and other or unspecified origins. These findings contribute to the understanding of the complex interplay between mental health, immune function, and female reproductive health. Additionally, to ensure the reliability and stability of our findings, we utilized recently published comprehensive GWAS data and performed multiple sensitivity analyses. These analyses further support the consistency and robustness of our results.

However, there are some limitations to consider. First, the inclusion of individuals of exclusively European ancestry restricts the generalizability of our findings to other racial or ethnic groups. Factors such as genetic variations, lifestyle, and environmental exposures may differ across populations, affecting the applicability of our results to other settings. Additionally, as for the PGC, we do not have the specific breakdown by sex for the MDD diagnosis. The limitations in the available sex-specific data, especially from the PGC, mean that we cannot provide an exact count of women diagnosed with MDD at the time of data collection for the entire study population. Subsequently, while we aimed to provide as much detail as possible, some of the databases we used did not fully provide specific information, such as exact ages or detailed infertility diagnoses and current MDD diagnoses. Then, our study relied on genetic data, which may be susceptible to confounding factors. While we employed MR to account for confounding, there may still be unmeasured or residual confounding that could influence our conclusions. Finally, the mechanisms underlying the association between MDD, IL-18, and female infertility were not fully elucidated. Further research is needed to explore these mechanisms and establish the therapeutic implications of our findings.

## 5. Conclusion

In conclusion, our study suggests a potential role of IL-18 as a partial mediator in the association between MDD and female infertility, particularly in cases with cervical, vaginal, and other or unspecified origin. These findings indicate a possible impact of MDD and IL-18 on female reproductive health. The complexity of factors influencing both MDD and infertility necessitates a careful interpretation of these associations. Given these findings, exploring anti-inflammatory interventions as adjunctive treatments for MDD-related infertility may be beneficial. Further research is needed to understand the exact mechanisms and develop targeted treatments that address the complex interplay of psychological and immunological factors in female infertility.

## Figures and Tables

**Table 1:** The bidirectional MR analysis and sensitivity analyses between MDD on IL-18 levels.

Exposure	Outcome	Methods	*β* (95% Cl)	OR (95% Cl)	*P*	Heterogeneity test				Horizontal pleiotropy test	
						MR-Egger		IVW		Egger-intercept	*P*
						Cochran's *Q*	*P*	Cochran's *Q*	*P*		
MDD	IL-18	IVW	−0.40 (−0.69 to −0.11)	0.67 (0.50–0.90)	7.09 × 10^−3^	41.62	0.32	41.71	0.35	−7.38 × 10^−3^	0.79
		MR-Egger	−0.15 (−1.97−1.65)	0.86 (0.14–5.19)	0.87						
		Weighted median	−0.33 (−0.76−0.08)	0.72 (0.47–1.08)	0.11						

IL-18	MDD	IVW	−2.52 × 10^−3^ (−0.03−0.02)	1.00 (0.97–1.02)	0.84	1.31	0.25	1.90	0.39	−0.03	0.62
		MR-Egger	0.12 (−0.24−0.46)	1.12 (0.79–1.59)	0.63						
		Weighted median	−2.60 × 10^−3^ (−0.03−0.03)	1.00 (0.97–1.03)	0.86						

*Abbreviations*. MDD, major depressive disorder; IL-18, interleukin-18; MR, Mendelian randomization; IVW, inverse variance-weighted; OR, odds ratio; Cl, confidence interval.

## Data Availability

The data that support the findings of this study are available in the IEU OpenGWAS project at https://gwas.mrcieu.ac.uk/, reference number: ieu-b-102, ebi-a-GCST004441. These data of reproductive health are available in FinnGen at https://www.finngen.fi. The analysis codes are available from the corresponding author upon reasonable request.

## Supplementary Materials

Supplementary 1 Table 1: the detailed information of MDD and IL-18 as IVs in the study.

Supplementary 2 Table 2: the basic information on the GWAS of exposure and outcome.

Supplementary 3 Table 3: the univariable MR analysis of MDD and IL-18 on reproductive health outcomes using the MR-Egger and weighted median methods.

Supplementary 4 Table 4: the multivariable MR analysis of MDD and IL-18 on reproductive health outcomes using the MR-Egger, weighted median, and MR-Lasso methods.

Supplementary 5 Table 5: the heterogeneity test and horizontal pleiotropy test for MDD and IL-18 on reproductive health outcomes using univariable MR analysis.

Supplementary 6 Table 6: the heterogeneity test and horizontal pleiotropy test for MDD and IL-18 on reproductive health outcomes using multivariable MR analysis.

Supplementary 7 Figure 1: the scatter plot of SNPs associated with MDD and infertility: (a) female infertility, (b) female infertility with cervical, vaginal, other or unspecified origin, and (c) female infertility with tubal origin, female infertility associated with anovulation. SNPs, single nucleotide polymorphisms; MDD, major depressive disorder.

Supplementary 8 Figure 2: the scatter plot of SNPs associated with MDD and infertility: (a) female infertility related to endometriosis, (b) female infertility with PCOS, and (c) male infertility. SNPs, single nucleotide polymorphisms; MDD, major depressive disorder; PCOS, polycystic ovary syndrome.

Supplementary 9 Figure 3: the scatter plot of SNPs associated with IL-18 levels and infertility: (a) female infertility, (b) female infertility with cervical, vaginal, other or unspecified origin, (c) female infertility with tubal origin, and (d) female infertility associated with anovulation. SNPs, single nucleotide polymorphisms; IL-18, interleukin-18.

Supplementary 10 Figure 4: the scatter plot of SNPs associated with IL-18 levels and infertility: (a) female infertility related to endometriosis, (b) female infertility with PCOS, and (c) male infertility. SNPs, single nucleotide polymorphisms; IL-18, interleukin-18; PCOS, polycystic ovary syndrome.

Supplementary 11 Figure 5: the funnel plot of SNPs associated with MDD and infertility: (a) female infertility, (b) female infertility with cervical, vaginal, other or unspecified origin, (c) female infertility with tubal origin, and (d) female infertility associated with anovulation. SNPs, single nucleotide polymorphisms; MDD, major depressive disorder.

Supplementary 12 Figure 6: the funnel plot of SNPs associated with MDD and infertility: (a) female infertility related to endometriosis, (b) female infertility with PCOS, and (c) male infertility. SNPs, single nucleotide polymorphisms; MDD, major depressive disorder; PCOS, polycystic ovary syndrome.

Supplementary 13 Figure 7: the funnel plot of SNPs associated with IL-18 levels and infertility: (a) female infertility, (b) female infertility with cervical, vaginal, other or unspecified origin, (c) female infertility with tubal origin, and (d) female infertility associated with anovulation. SNPs, single nucleotide polymorphisms; IL-18, interleukin-18.

Supplementary 14 Figure 8: the funnel plot of SNPs associated with IL-18 levels and infertility: (a) female infertility related to endometriosis, (b) female infertility with PCOS, and (c) male infertility. SNPs, single nucleotide polymorphisms; IL-18, interleukin-18; PCOS, polycystic ovary syndrome.

Supplementary 15 Figure 9: the leave-one-out test plot of the causal association between MDD and infertility: (a) female infertility, (b) female infertility with cervical, vaginal, other or unspecified origin, (c) female infertility with tubal origin, and (d) female infertility associated with anovulation. MDD, major depressive disorder.

Supplementary 16 Figure 10: the leave-one-out test plot of the causal association between MDD and infertility: (a) female infertility related to endometriosis, (b) female infertility with PCOS, and (c) male infertility. SNPs, single nucleotide polymorphisms; MDD, major depressive disorder; PCOS, polycystic ovary syndrome.

Supplementary 17 Figure 11: the leave-one-out test plot of the causal association between IL-18 levels and infertility: (a) female infertility, (b) female infertility with cervical, vaginal, other or unspecified origin, (c) female infertility with tubal origin, and (d) female infertility associated with anovulation. IL-18, interleukin-18.

Supplementary 18 Figure 12: the leave-one-out test plot of the causal association between IL-18 levels and infertility: (a) female infertility related to endometriosis, (b) female infertility with PCOS, and (c) male infertility. SNPs, single nucleotide polymorphisms; IL-18, interleukin-18; PCOS, polycystic ovary syndrome.
