# Supplementary material for: Perspective Exploring Novel Associations of IL-18 Levels as a Mediator of the Causal Links between Major Depression and Reproductive Health
Source: Depress Anxiety. 2024 Aug 5;2024:9234876. doi: 10.1155/2024/9234876 (PMC11918975; doi:10.1155/2024/9234876)
Supplement: Supplementary 1 — Table 1: the detailed information of MDD and IL-18 as IVs in the study. [file 9234876.f1.docx]

Table S1. The detailed information of MDD and IL-18 as IVs in the study.

| Exposure | ID | SNP | Chr | Pos | Effect allele | Other allele | Beta | SE | P value | Eaf |
| --- | --- | --- | --- | --- | --- | --- | --- | --- | --- | --- |
| Major depression | ieu-b-102 | rs2568958 | 1 | 72765116 | A | G | 0.0382 | 0.0044 | 2.90E-18 | 0.6042 |
| Major depression | ieu-b-102 | rs10913112 | 1 | 175913828 | T | C | -0.0262 | 0.0045 | 4.53E-09 | 0.378 |
| Major depression | ieu-b-102 | rs17641524 | 1 | 197704717 | T | C | -0.03 | 0.0053 | 1.50E-08 | 0.2101 |
| Major depression | ieu-b-102 | rs354155 | 1 | 49675276 | C | G | -0.0449 | 0.0075 | 1.75E-09 | 0.0923 |
| Major depression | ieu-b-102 | rs7538938 | 1 | 67132262 | C | T | 0.0251 | 0.0043 | 7.29E-09 | 0.5599 |
| Major depression | ieu-b-102 | rs4141983 | 1 | 18122009 | C | T | -0.0264 | 0.0046 | 9.69E-09 | 0.326 |
| Major depression | ieu-b-102 | rs2111592 | 2 | 208049581 | A | G | 0.0263 | 0.0046 | 1.35E-08 | 0.3141 |
| Major depression | ieu-b-102 | rs72948506 | 2 | 212618440 | A | G | 0.0265 | 0.0047 | 1.71E-08 | 0.2975 |
| Major depression | ieu-b-102 | rs35469634 | 3 | 158171455 | G | A | -0.0241 | 0.0044 | 3.28E-08 | 0.5774 |
| Major depression | ieu-b-102 | rs843812 | 3 | 61255413 | A | G | 0.0248 | 0.0044 | 1.41E-08 | 0.4117 |
| Major depression | ieu-b-102 | rs9831648 | 3 | 49214303 | T | G | -0.0292 | 0.0052 | 1.59E-08 | 0.7739 |
| Major depression | ieu-b-102 | rs66511648 | 3 | 117515519 | C | T | 0.0297 | 0.0048 | 6.03E-10 | 0.284 |
| Major depression | ieu-b-102 | rs76954012 | 3 | 115977242 | A | T | 0.0412 | 0.0074 | 2.41E-08 | 0.0931 |
| Major depression | ieu-b-102 | rs30266 | 5 | 103972357 | A | G | 0.0366 | 0.0046 | 1.43E-15 | 0.3271 |
| Major depression | ieu-b-102 | rs247910 | 5 | 87630769 | G | A | 0.0237 | 0.0043 | 4.71E-08 | 0.457 |
| Major depression | ieu-b-102 | rs7725715 | 5 | 164487555 | A | G | 0.029 | 0.0043 | 1.61E-11 | 0.5343 |
| Major depression | ieu-b-102 | rs150186873 | 6 | 27182377 | C | A | 0.0704 | 0.012 | 4.51E-09 | 0.0327 |
| Major depression | ieu-b-102 | rs2232423 | 6 | 28366151 | G | A | -0.062 | 0.007 | 1.14E-18 | 0.1056 |
| Major depression | ieu-b-102 | rs9364755 | 6 | 165117329 | G | A | 0.0283 | 0.0051 | 3.49E-08 | 0.2262 |
| Major depression | ieu-b-102 | rs2214123 | 6 | 67000001 | G | A | -0.0261 | 0.0045 | 8.56E-09 | 0.6466 |
| Major depression | ieu-b-102 | rs2876520 | 6 | 142996618 | G | C | 0.026 | 0.0043 | 2.24E-09 | 0.4688 |
| Major depression | ieu-b-102 | rs2522831 | 7 | 82448100 | C | T | 0.024 | 0.0043 | 2.11E-08 | 0.4739 |
| Major depression | ieu-b-102 | rs4730387 | 7 | 109100414 | A | T | 0.0238 | 0.0043 | 4.12E-08 | 0.4659 |
| Major depression | ieu-b-102 | rs150346963 | 7 | 117625599 | T | C | 0.0283 | 0.0044 | 1.16E-10 | 0.4118 |
| Major depression | ieu-b-102 | rs3807865 | 7 | 12250402 | A | G | 0.031 | 0.0044 | 1.09E-12 | 0.4105 |
| Major depression | ieu-b-102 | rs10235664 | 7 | 2086814 | C | T | -0.027 | 0.0049 | 4.68E-08 | 0.2529 |
| Major depression | ieu-b-102 | rs59082935 | 7 | 38724868 | T | C | 0.0363 | 0.0066 | 3.07E-08 | 0.1342 |
| Major depression | ieu-b-102 | rs62535714 | 9 | 37182655 | A | G | 0.0339 | 0.0058 | 4.69E-09 | 0.1639 |
| Major depression | ieu-b-102 | rs1931388 | 9 | 11203149 | G | A | -0.0295 | 0.0044 | 1.68E-11 | 0.4042 |
| Major depression | ieu-b-102 | rs59283172 | 9 | 25232978 | A | G | -0.039 | 0.007 | 2.41E-08 | 0.1081 |
| Major depression | ieu-b-102 | rs2418449 | 9 | 119731359 | C | T | -0.0281 | 0.0048 | 4.25E-09 | 0.281 |
| Major depression | ieu-b-102 | rs1021363 | 10 | 106610839 | G | A | -0.03 | 0.0045 | 2.29E-11 | 0.6434 |
| Major depression | ieu-b-102 | rs198457 | 11 | 61471678 | T | C | -0.0315 | 0.0056 | 1.90E-08 | 0.1886 |
| Major depression | ieu-b-102 | rs4497414 | 11 | 88756779 | C | T | 0.0291 | 0.0044 | 2.93E-11 | 0.44 |
| Major depression | ieu-b-102 | rs4936276 | 11 | 113365141 | C | G | 0.0278 | 0.0044 | 3.57E-10 | 0.622 |
| Major depression | ieu-b-102 | rs61914045 | 12 | 52352301 | A | G | 0.0309 | 0.0054 | 7.96E-09 | 0.2034 |
| Major depression | ieu-b-102 | rs9529218 | 13 | 31790053 | T | C | -0.034 | 0.0054 | 2.23E-10 | 0.2031 |
| Major depression | ieu-b-102 | rs9536381 | 13 | 53860655 | T | C | 0.0255 | 0.0046 | 2.62E-08 | 0.3259 |
| Major depression | ieu-b-102 | rs508502 | 13 | 80921519 | T | C | -0.0264 | 0.0048 | 3.56E-08 | 0.2992 |
| Major depression | ieu-b-102 | rs1950829 | 14 | 42097937 | G | A | -0.0297 | 0.0043 | 4.74E-12 | 0.5173 |
| Major depression | ieu-b-102 | rs754287 | 14 | 103997525 | A | T | -0.0289 | 0.0045 | 1.31E-10 | 0.3664 |
| Major depression | ieu-b-102 | rs7152906 | 14 | 75125540 | C | T | 0.0258 | 0.0043 | 1.87E-09 | 0.5196 |
| Major depression | ieu-b-102 | rs28541419 | 15 | 88945878 | G | C | -0.0292 | 0.0052 | 1.76E-08 | 0.2308 |
| Major depression | ieu-b-102 | rs12919291 | 16 | 13800430 | C | G | 0.0327 | 0.0055 | 3.09E-09 | 0.1884 |
| Major depression | ieu-b-102 | rs4799949 | 18 | 35155910 | T | C | -0.0292 | 0.0046 | 1.40E-10 | 0.6684 |
| Major depression | ieu-b-102 | rs12967143 | 18 | 53099012 | C | G | -0.0345 | 0.0047 | 2.53E-13 | 0.7012 |
| Major depression | ieu-b-102 | rs7241572 | 18 | 77580712 | A | G | 0.0323 | 0.0054 | 2.43E-09 | 0.2047 |
| Major depression | ieu-b-102 | rs1367635 | 18 | 50861409 | C | T | 0.0253 | 0.0043 | 4.35E-09 | 0.5148 |
| Major depression | ieu-b-102 | rs13037326 | 20 | 44692598 | T | C | 0.031 | 0.0049 | 2.40E-10 | 0.2597 |
| Interleukin-18 levels | ebi-a-GCST004441 | rs385076 | 2 | 32489851 | C | T | 0.2432 | 0.0248 | 1.66E-22 | NA |
| Interleukin-18 levels | ebi-a-GCST004441 | rs115267715 | 5 | 68535015 | T | C | 0.4508 | 0.08 | 1.72E-08 | NA |
| Interleukin-18 levels | ebi-a-GCST004441 | rs17229943 | 5 | 68682536 | C | A | 0.312 | 0.0463 | 1.62E-11 | NA |
| Interleukin-18 levels | ebi-a-GCST004441 | rs71478720 | 11 | 112009605 | T | C | -0.2669 | 0.0276 | 3.71E-22 | NA |

Abbreviations: MDD: Major depressive disorder; IL-18: Interleukin-18; IVs: Instrumental variables.
