# Supplementary material for: Perspective Exploring Novel Associations of IL-18 Levels as a Mediator of the Causal Links between Major Depression and Reproductive Health
Source: Depress Anxiety. 2024 Aug 5;2024:9234876. doi: 10.1155/2024/9234876 (PMC11918975; doi:10.1155/2024/9234876)
Supplement: Supplementary 2 — Table 2: the basic information on the GWAS of exposure and outcome. [file 9234876.f2.docx]

Table S2. The basic information on the GWAS of exposure and outcome.

| GWAS Source | Author | Year | Trait | Number of sample Size | Number of cases and Controls | GWAS ID | Consortium | Population | Number of SNPs |
| --- | --- | --- | --- | --- | --- | --- | --- | --- | --- |
| PMID: 30718901 | Howard DM | 2019 | Major depression | 500,199 | 170,756 cases and 329,443 controls | ieu-b-102 | PGC and UK biobank | European | 8,483,301 |
| PMID: 27989323 | Ahola-Olli AV | 2016 | Interleukin-18 levels | 3,636 | NA | ebi-a-GCST004441 | The Cardiovascular Risk in Young Finns Study and The National FINRISK Study | European | 9,785,222 |
| NA | NA | 2022 | Female infertility | 120,706 | 13,142 cases and 107,564 controls | NA | The FinnGen Biobank | European | 20,140,101 |
| NA | NA | 2022 | [Female infertility, cervigal, vaginal, other or unspecified origin](https://r9.finngen.fi/pheno/N14_FIOTHNAS) | 118,912 | 11,348 cases and 107,564 controls | NA | The FinnGen Biobank | European | 20,139,164 |
| NA | NA | 2022 | Female infertility, tubal origin | 109,047 | 1,483 cases and 107,564 controls | NA | The FinnGen Biobank | European | 20,132,531 |
| NA | NA | 2022 | Female infertility, associated with anovulation | 110,005 | 2,441 cases and 107,564 controls | NA | The FinnGen Biobank | European | 20,133,215 |
| NA | NA | 2022 | Endometriosis diagnosis and infertility diagnosis occurring together | 204,241 | 3,206 cases and 201,035 controls | NA | The FinnGen Biobank | European | 20,161,750 |
| NA | NA | 2022 | [Polycystic ovarian syndrome, consortium definition](https://r9.finngen.fi/pheno/E4_PCOS_CONCORTIUM) | 210,870 | 31,548 cases and 179,322 controls | NA | The FinnGen Biobank | European | 20,162,430 |
| NA | NA | 2022 | [Male infertility](https://r9.finngen.fi/pheno/N14_MALEINFERT) | 120,568 | 1,271 cases and 119,297 controls | NA | The FinnGen Biobank | European | 20,139,487 |

Abbreviations: MDD: Major depressive disorder; IL-18: Interleukin-18; MR: Mendelian randomization; SNP: Single nucleotide polymorphisms; GWAS: Genome-wide association studies; PGC: Psychiatric Genomics Consortium; NA: Not applicable.
