# Supplementary material for: Perspective Exploring Novel Associations of IL-18 Levels as a Mediator of the Causal Links between Major Depression and Reproductive Health
Source: Depress Anxiety. 2024 Aug 5;2024:9234876. doi: 10.1155/2024/9234876 (PMC11918975; doi:10.1155/2024/9234876)
Supplement: Supplementary 3 — Table 3: the univariable MR analysis of MDD and IL-18 on reproductive health outcomes using the MR-Egger and weighted median methods. [file 9234876.f3.docx]

Table S3. The univariable MR analysis of MDD and IL-18 on reproductive health outcomes using the MR-Egger and weighted median methods.

| Exposure | Outcomes | MR-Egger | | | Weighted median | | |
| --- | --- | --- | --- | --- | --- | --- | --- |
|  |  | β | OR (95%Cl) | *p* | β | OR (95%Cl) | *p* |
| MDD | Female infertility | -0.42 | 0.66 (0.25-1.74) | 0.40 | 0.30 | 1.35 (1.08-1.68) | 0.01 |
|  | —Cervical, vaginal, other or unspecified origin | -0.53 | 0.59 (0.21-1.66) | 0.32 | 0.29 | 1.33 (1.05-1.68) | 0.02 |
|  | —Tubal origin | -0.15 | 0.86 (0.05-15.38) | 0.92 | 0.39 | 1.47 (0.75-2.88) | 0.26 |
|  | —Anovulation associated | 1.11 | 3.03 (0.31-29.75) | 0.35 | 0.13 | 1.14 (0.69-1.87) | 0.62 |
|  | —Endometriosis related | -0.42 | 0.66 (0.09-4.90) | 0.68 | -0.08 | 0.92 (0.60-1.43) | 0.72 |
|  | —PCOS | -0.34 | 0.71 (0.36-1.40) | 0.33 | 0.22 | 1.25 (1.07-1.46) | 0.01 |
|  | Male infertility | 1.43 | 4.16 (0.21-82.86) | 0.36 | -0.21 | 0.81 (0.41-1.58) | 0.54 |
| IL-18 | Female infertility | -0.25 | 0.78 (0.51-1.18) | 0.36 | -0.09 | 0.91 (0.84-0.99) | 0.02 |
|  | —Cervical, vaginal, other or unspecified origin | -0.21 | 0.81 (0.52-1.27) | 0.46 | -0.08 | 0.92 (0.84-1.00) | 0.06 |
|  | —Tubal origin | 0.50 | 1.65 (0.50-5.41) | 0.50 | 0.01 | 1.01 (0.81-1.25) | 0.95 |
|  | —Anovulation associated | -0.30 | 0.74 (0.21-2.60) | 0.68 | -0.07 | 0.93 (0.77-1.12) | 0.45 |
|  | —Endometriosis related | -0.03 | 0.97 (0.26-3.62) | 0.97 | -0.08 | 0.92 (0.78-1.09) | 0.33 |
|  | —PCOS | -0.20 | 0.82 (0.61-1.08) | 0.29 | -0.02 | 0.98 (0.92-1.03) | 0.37 |
|  | Male infertility | 0.76 | 2.13 (0.62-7.36) | 0.35 | 0.03 | 1.03 (0.80-1.32) | 0.80 |

Abbreviations: MDD: Major depressive disorder; IL-18: Interleukin-18; MR: Mendelian randomization; PCOS: Polycystic ovary syndrome; OR: Odds ratio; Cl: Confidence interval.
