# Supplementary material for: Perspective Exploring Novel Associations of IL-18 Levels as a Mediator of the Causal Links between Major Depression and Reproductive Health
Source: Depress Anxiety. 2024 Aug 5;2024:9234876. doi: 10.1155/2024/9234876 (PMC11918975; doi:10.1155/2024/9234876)
Supplement: Supplementary 4 — Table 4: the multivariable MR analysis of MDD and IL-18 on reproductive health outcomes using the MR-Egger, weighted median, and MR-Lasso methods. [file 9234876.f4.docx]

Table S4. The multivariable MR analysis of MDD and IL-18 on reproductive health outcomes using the MR-Egger, weighted median and MR- Lasso methods.

| Exposure | Outcomes | Methods | β | OR (95%Cl) | *p* |
| --- | --- | --- | --- | --- | --- |
| MDD adjusted for IL-18 | Female infertility | Multivariable MR-Egger | -0.09 | 0.91(0.52-1.60) | 0.75 |
|  | —Cervical, vaginal, other or unspecified origin |  | -0.24 | 0.78(0.43-1.42) | 0.42 |
|  | —Tubal origin |  | -0.37 | 0.69(0.14-3.34) | 0.64 |
|  | —Anovulation associated |  | 1.28 | 3.61(1.00-13.07) | 0.05 |
|  | —Endometriosis related |  | 0.50 | 1.65(0.51-5.35) | 0.40 |
|  | —PCOS |  | -0.13 | 0.88(0.59-1.30) | 0.51 |
|  | Male infertility |  | 0.46 | 1.59(0.29-8.86) | 0.60 |
| IL-18 adjusted for MDD | Female infertility |  | -0.07 | 0.94(0.88-1.00) | 0.04 |
|  | —Cervical, vaginal, other or unspecified origin |  | -0.08 | 0.93(0.87-0.99) | 0.03 |
|  | —Tubal origin |  | 0.05 | 1.05(0.88-1.26) | 0.61 |
|  | —Anovulation associated |  | -0.08 | 0.93(0.80-1.07) | 0.32 |
|  | —Endometriosis related |  | -0.09 | 0.91(0.80-1.04) | 0.19 |
|  | —PCOS |  | -0.03 | 0.97(0.93-1.02) | 0.23 |
|  | Male infertility |  | 0.06 | 1.06(0.87-1.29) | 0.54 |
| MDD adjusted for IL-18 | Female infertility | Multivariable weighted median | 0.28 | 1.32(1.07-1.64) | 0.01 |
|  | —Cervical, vaginal, other or unspecified origin |  | 0.24 | 1.28(1.02-1.60) | 0.04 |
|  | —Tubal origin |  | 0.28 | 1.32(0.73-2.37) | 0.36 |
|  | —Anovulation associated |  | 0.20 | 1.22(0.75-1.99) | 0.42 |
|  | —Endometriosis related |  | 0.03 | 1.03(0.68-1.57) | 0.87 |
|  | —PCOS |  | 0.20 | 1.22(1.06-1.41) | 0.01 |
|  | Male infertility |  | 0.07 | 1.07(0.57-2.01) | 0.83 |
| IL-18 adjusted for MDD | Female infertility |  | -0.04 | 0.96(0.88-1.06) | 0.43 |
|  | —Cervical, vaginal, other or unspecified origin |  | -0.09 | 0.92(0.84-1.00) | 0.05 |
|  | —Tubal origin |  | 0.08 | 1.08(0.85-1.38) | 0.51 |
|  | —Anovulation associated |  | 0.10 | 1.11(0.88-1.4) | 0.39 |
|  | —Endometriosis related |  | -0.03 | 0.98(0.81-1.18) | 0.79 |
|  | —PCOS |  | -0.02 | 0.98(0.93-1.03) | 0.44 |
|  | Male infertility |  | 0.03 | 1.03(0.80-1.35) | 0.80 |
| MDD adjusted for IL-18 | Female infertility | Multivariable MR-Lasso | 0.18 | 1.19(1.03-1.38) | 0.02 |
|  | —Cervical, vaginal, other or unspecified origin |  | 0.16 | 1.18(1.01-1.38) | 0.04 |
|  | —Tubal origin |  | 0.16 | 1.17(0.78-1.76) | 0.45 |
|  | —Anovulation associated |  | 0.34 | 1.40(1.00-1.97) | 0.05 |
|  | —Endometriosis related |  | 0.05 | 1.06(0.79-1.41) | 0.71 |
|  | —PCOS |  | 0.18 | 1.20(1.08-1.33) | 1.00E-03 |
|  | Male infertility |  | 0.33 | 1.40(0.89-2.17) | 0.14 |
| IL-18 adjusted for MDD | Female infertility |  | -0.07 | 0.93(0.87-0.99) | 0.02 |
|  | —Cervical, vaginal, other or unspecified origin |  | -0.08 | 0.92(0.86-0.98) | 0.01 |
|  | —Tubal origin |  | 0.04 | 1.04(0.87-1.24) | 0.69 |
|  | —Anovulation associated |  | -0.06 | 0.95(0.82-1.10) | 0.46 |
|  | —Endometriosis related |  | 0.00 | 1.00(0.86-1.15) | 0.96 |
|  | —PCOS |  | -0.03 | 0.97(0.92-1.01) | 0.14 |
|  | Male infertility |  | 0.06 | 1.07(0.88-1.29) | 0.52 |

Abbreviations: MDD: Major depressive disorder; IL-18: Interleukin-18; MR: Mendelian randomization; PCOS: Polycystic ovary syndrome; OR: Odds ratio; Cl: Confidence interval.
