# Supplementary material for: Perspective Exploring Novel Associations of IL-18 Levels as a Mediator of the Causal Links between Major Depression and Reproductive Health
Source: Depress Anxiety. 2024 Aug 5;2024:9234876. doi: 10.1155/2024/9234876 (PMC11918975; doi:10.1155/2024/9234876)
Supplement: Supplementary 5 — Table 5: the heterogeneity test and horizontal pleiotropy test for MDD and IL-18 on reproductive health outcomes using univariable MR analysis. [file 9234876.f5.docx]

Table S5. The heterogeneity test and horizontal pleiotropy test for MDD and IL-18 on reproductive health outcomes using univariable MR analysis.

| Exposure | Outcome | Heterogeneity test | | | | Horizontal pleiotropy test | |
| --- | --- | --- | --- | --- | --- | --- | --- |
|  |  | MR-Egger | | IVW | |  |  |
|  |  | Cochran's Q | *p* | Cochran's Q | *p* | Egger-intercept | *p* |
| MDD | Female infertility | 34.62 | 0.58 | 36.39 | 0.54 | 0.02 | 0.19 |
|  | —Cervical, vaginal, other or unspecified origin | 33.46 | 0.64 | 35.60 | 0.58 | 0.02 | 0.15 |
|  | —Tubal origin | 40.98 | 0.30 | 41.04 | 0.34 | 0.01 | 0.34 |
|  | —Anovulation associated | 41.58 | 0.28 | 42.11 | 0.30 | -0.02 | 0.50 |
|  | —Endometriosis related | 42.98 | 0.23 | 43.08 | 0.26 | 0.01 | 0.77 |
|  | —PCOS | 41.05 | 0.30 | 44.12 | 0.23 | 0.02 | 0.10 |
|  | Male infertility | 34.53 | 0.59 | 35.19 | 0.60 | -0.04 | 0.42 |
| IL-18 | Female infertility | 0.97 | 0.62 | 1.61 | 0.66 | 0.05 | 0.51 |
|  | —Cervical, vaginal, other or unspecified origin | 0.39 | 0.82 | 0.64 | 0.89 | 0.03 | 0.67 |
|  | —Tubal origin | 0.82 | 0.66 | 1.55 | 0.67 | -0.14 | 0.48 |
|  | —Anovulation associated | 3.71 | 0.16 | 3.94 | 0.27 | 0.06 | 0.76 |
|  | —Endometriosis related | 5.30 | 0.07 | 5.33 | 0.15 | -0.02 | 0.93 |
|  | —PCOS | 0.83 | 0.66 | 2.30 | 0.51 | 0.05 | 0.35 |
|  | Male infertility | 0.76 | 0.68 | 2.20 | 0.53 | -0.21 | 0.35 |

Abbreviations: MDD: Major depressive disorder; IL-18: Interleukin-18; MR: Mendelian randomization; PCOS: Polycystic ovary syndrome; IVW: Inverse variance-weighted.
