# Supplementary material for: Perspective Exploring Novel Associations of IL-18 Levels as a Mediator of the Causal Links between Major Depression and Reproductive Health
Source: Depress Anxiety. 2024 Aug 5;2024:9234876. doi: 10.1155/2024/9234876 (PMC11918975; doi:10.1155/2024/9234876)
Supplement: Supplementary 6 — Table 6: the heterogeneity test and horizontal pleiotropy test for MDD and IL-18 on reproductive health outcomes using multivariable MR analysis. [file 9234876.f6.docx]

Table S6. The heterogeneity test and horizontal pleiotropy test for MDD and IL-18 on reproductive health outcomes using multivariable MR analysis.

| Exposure | Outcomes | Heterogeneity test | | Horizontal pleiotropy test | |
| --- | --- | --- | --- | --- | --- |
|  |  | Cochran's Q | *p* | Egger-intercept | *p* |
| MDD and IL-18 | Female infertility | 46.93 | 0.48 | 8.00E-03 | 0.34 |
|  | —Cervical, vaginal, other or unspecified origin | 48.45 | 0.41 | 0.01 | 0.17 |
|  | —Tubal origin | 47.99 | 0.43 | 0.01 | 0.50 |
|  | —Anovulation associated | 47.92 | 0.44 | -0.03 | 0.14 |
|  | —Endometriosis related | 48.15 | 0.43 | -0.02 | 0.30 |
|  | —PCOS | 46.23 | 0.50 | 9.00E-03 | 0.11 |
|  | Male infertility | 47.81 | 0.44 | -4.00E-03 | 0.88 |

Abbreviations: MDD: Major depressive disorder; IL-18: Interleukin-18; MR: Mendelian randomization; PCOS: Polycystic ovary syndrome.
