# Supplementary figures and images for: Perspective Exploring Novel Associations of IL-18 Levels as a Mediator of the Causal Links between Major Depression and Reproductive Health
Source: Depress Anxiety. 2024 Aug 5;2024:9234876. doi: 10.1155/2024/9234876 (PMC11918975; doi:10.1155/2024/9234876)

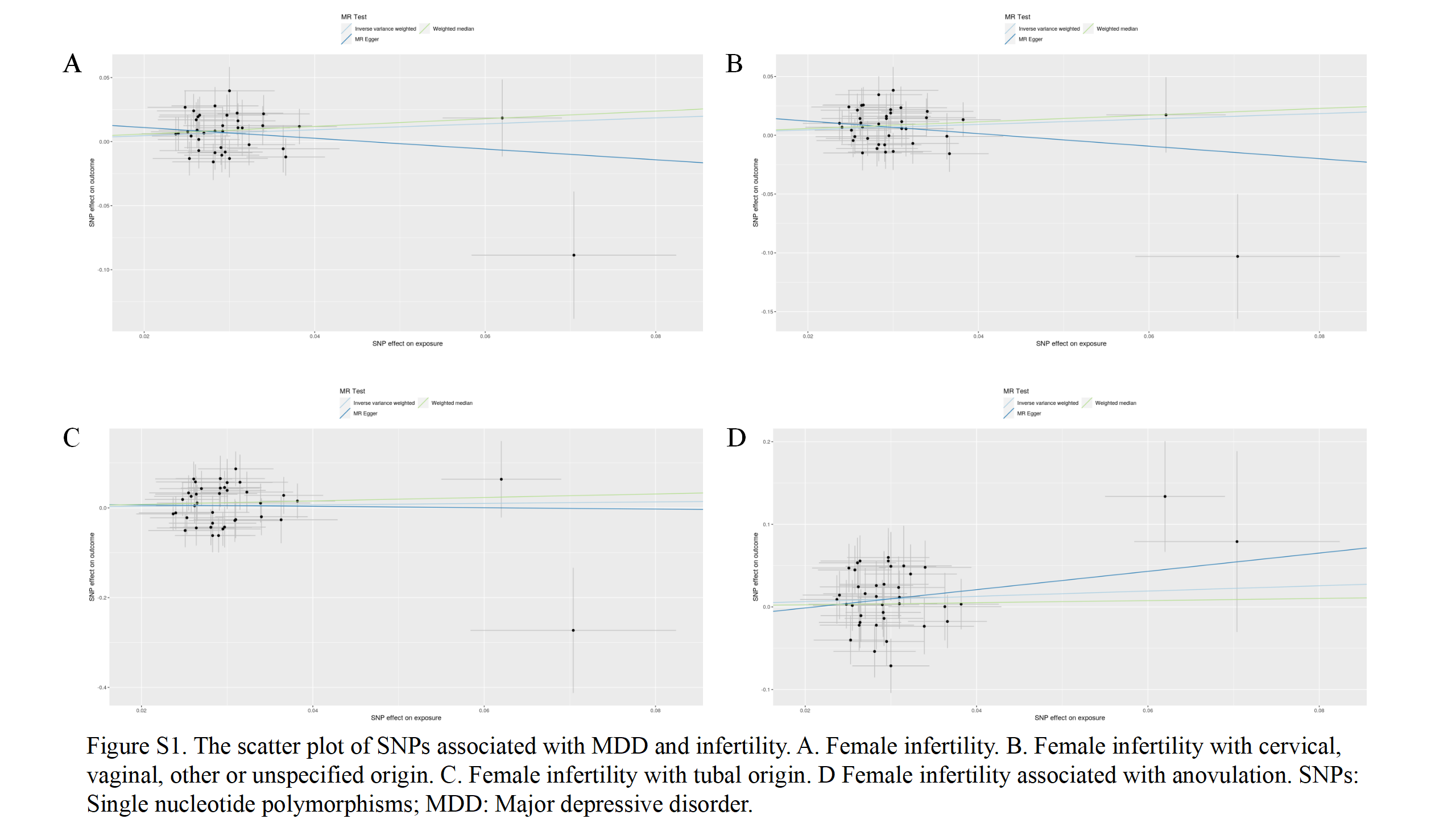

Supplement: Supplementary 7 — Figure 1: the scatter plot of SNPs associated with MDD and infertility: (a) female infertility, (b) female infertility with cervical, vaginal, other or unspecified origin, and (c) female infertility with tubal origin, female infertility associated with anovulation. SNPs, single nucleotide polymorphisms; MDD, major depressive disorder. [file 9234876.f7.tif]

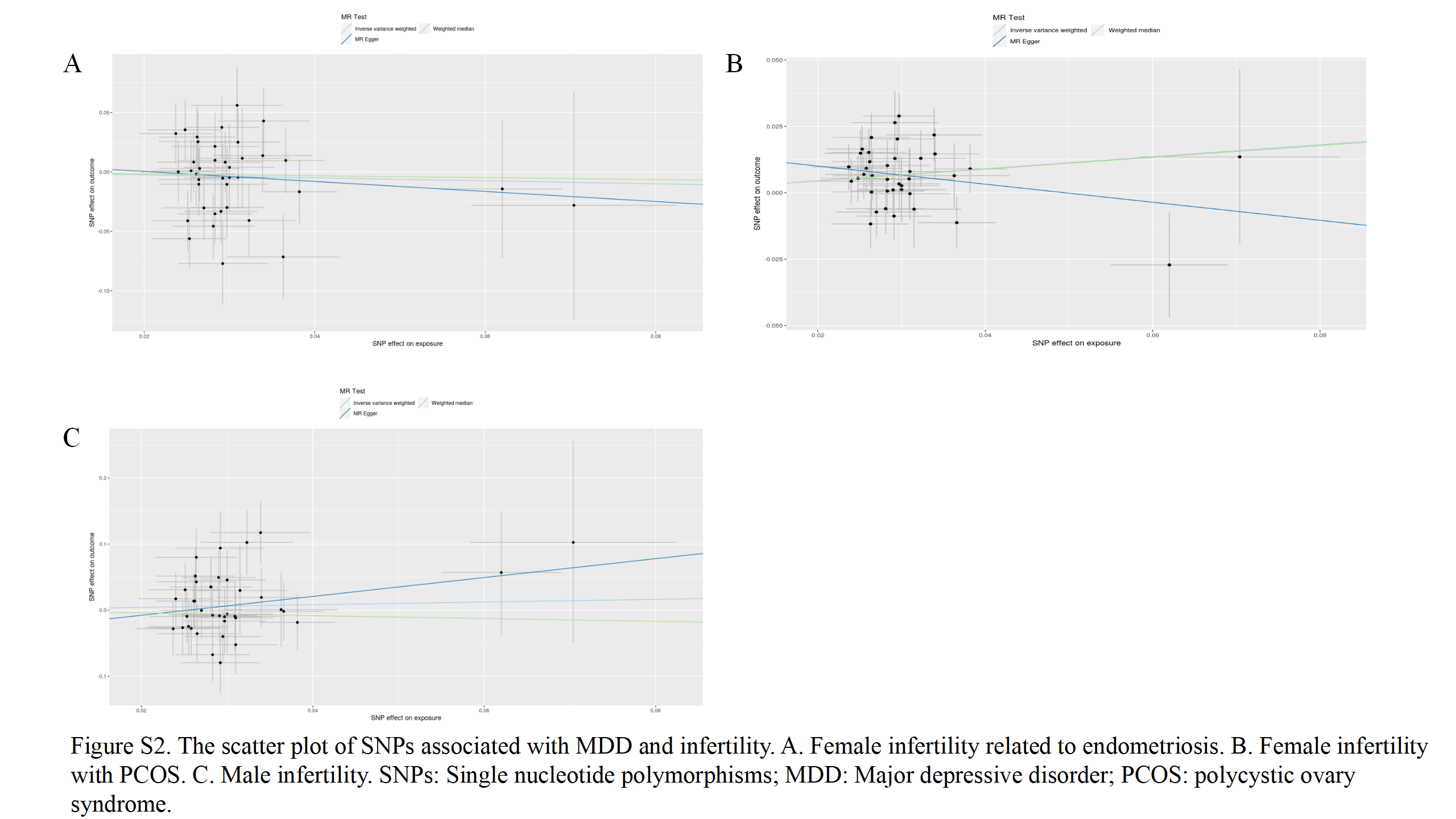

Supplement: Supplementary 8 — Figure 2: the scatter plot of SNPs associated with MDD and infertility: (a) female infertility related to endometriosis, (b) female infertility with PCOS, and (c) male infertility. SNPs, single nucleotide polymorphisms; MDD, major depressive disorder; PCOS, polycystic ovary syndrome. [file 9234876.f8.tif]

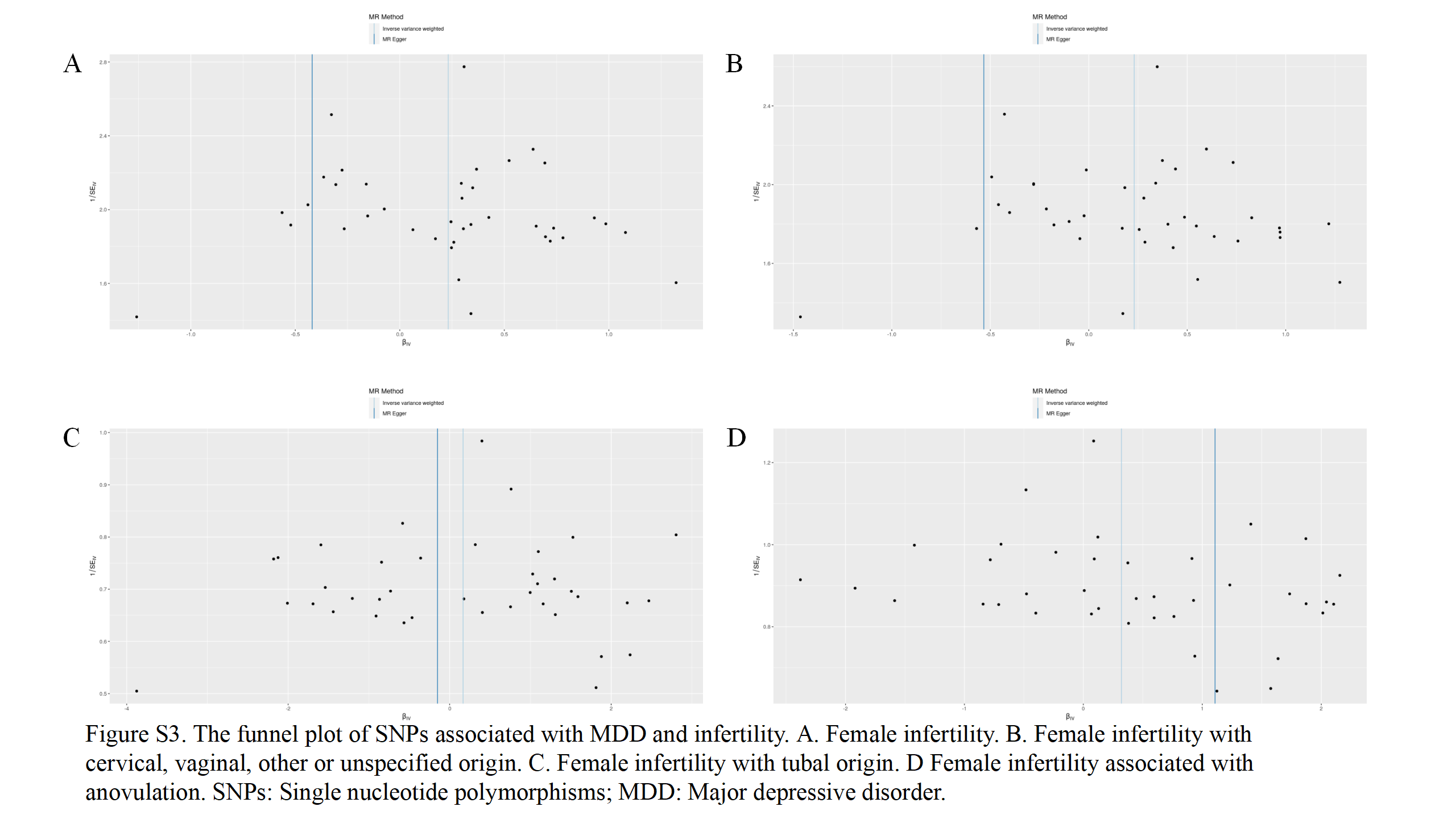

Supplement: Supplementary 9 — Figure 3: the scatter plot of SNPs associated with IL-18 levels and infertility: (a) female infertility, (b) female infertility with cervical, vaginal, other or unspecified origin, (c) female infertility with tubal origin, and (d) female infertility associated with anovulation. SNPs, single nucleotide polymorphisms; IL-18, interleukin-18. [file 9234876.f9.tif]

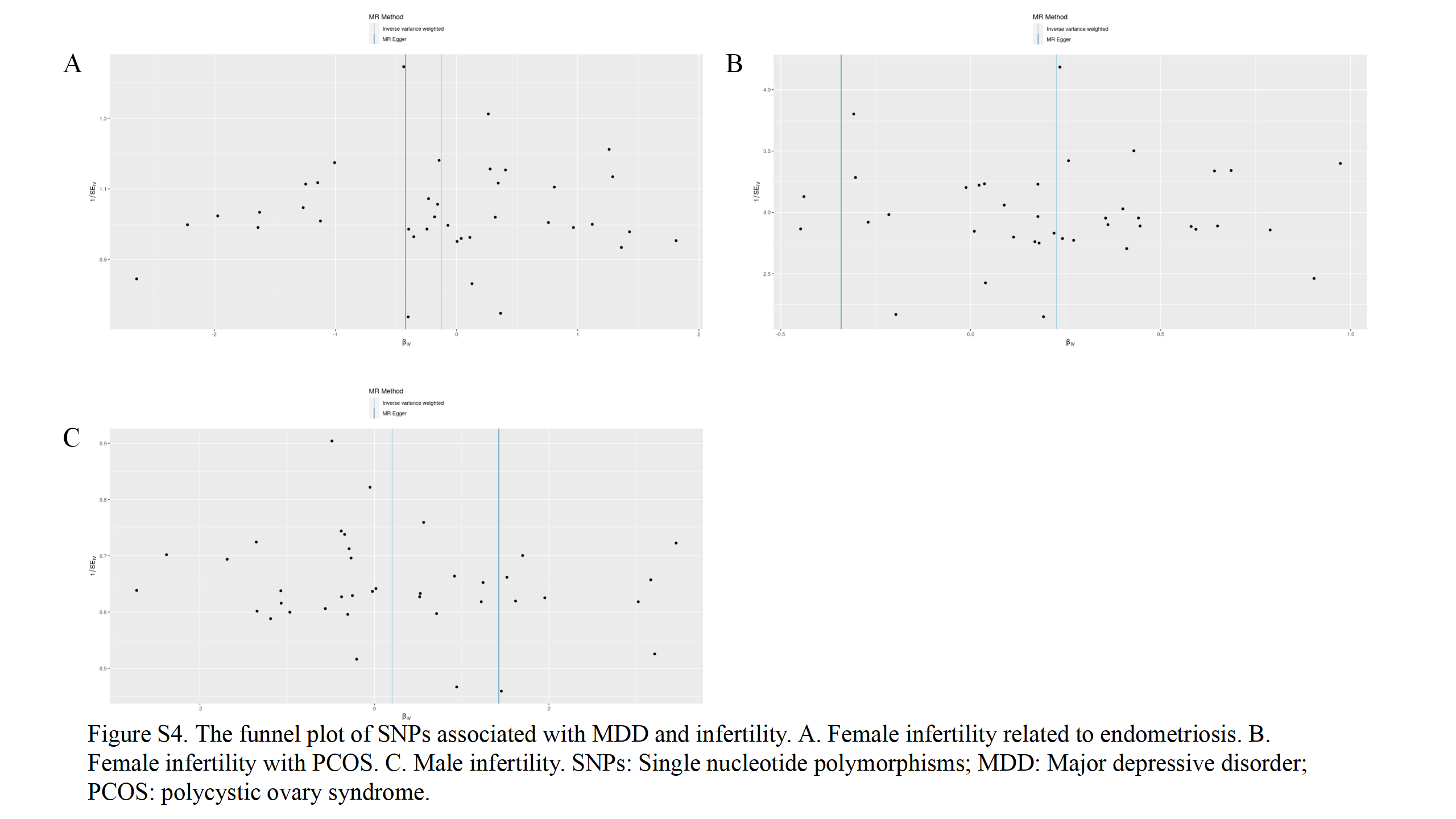

Supplement: Supplementary 10 — Figure 4: the scatter plot of SNPs associated with IL-18 levels and infertility: (a) female infertility related to endometriosis, (b) female infertility with PCOS, and (c) male infertility. SNPs, single nucleotide polymorphisms; IL-18, interleukin-18; PCOS, polycystic ovary syndrome. [file 9234876.f10.tif]

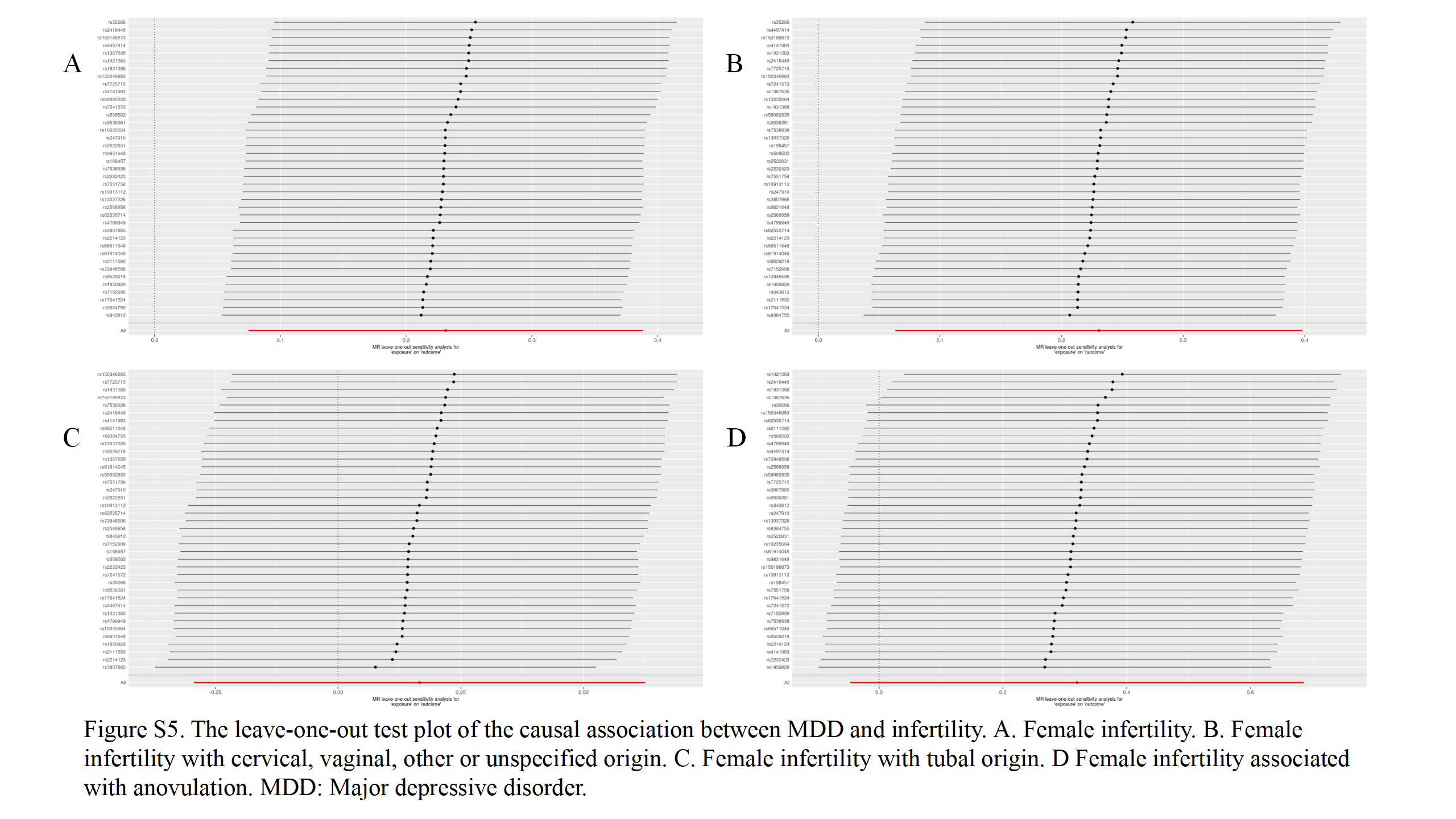

Supplement: Supplementary 11 — Figure 5: the funnel plot of SNPs associated with MDD and infertility: (a) female infertility, (b) female infertility with cervical, vaginal, other or unspecified origin, (c) female infertility with tubal origin, and (d) female infertility associated with anovulation. SNPs, single nucleotide polymorphisms; MDD, major depressive disorder. [file 9234876.f11.tif]

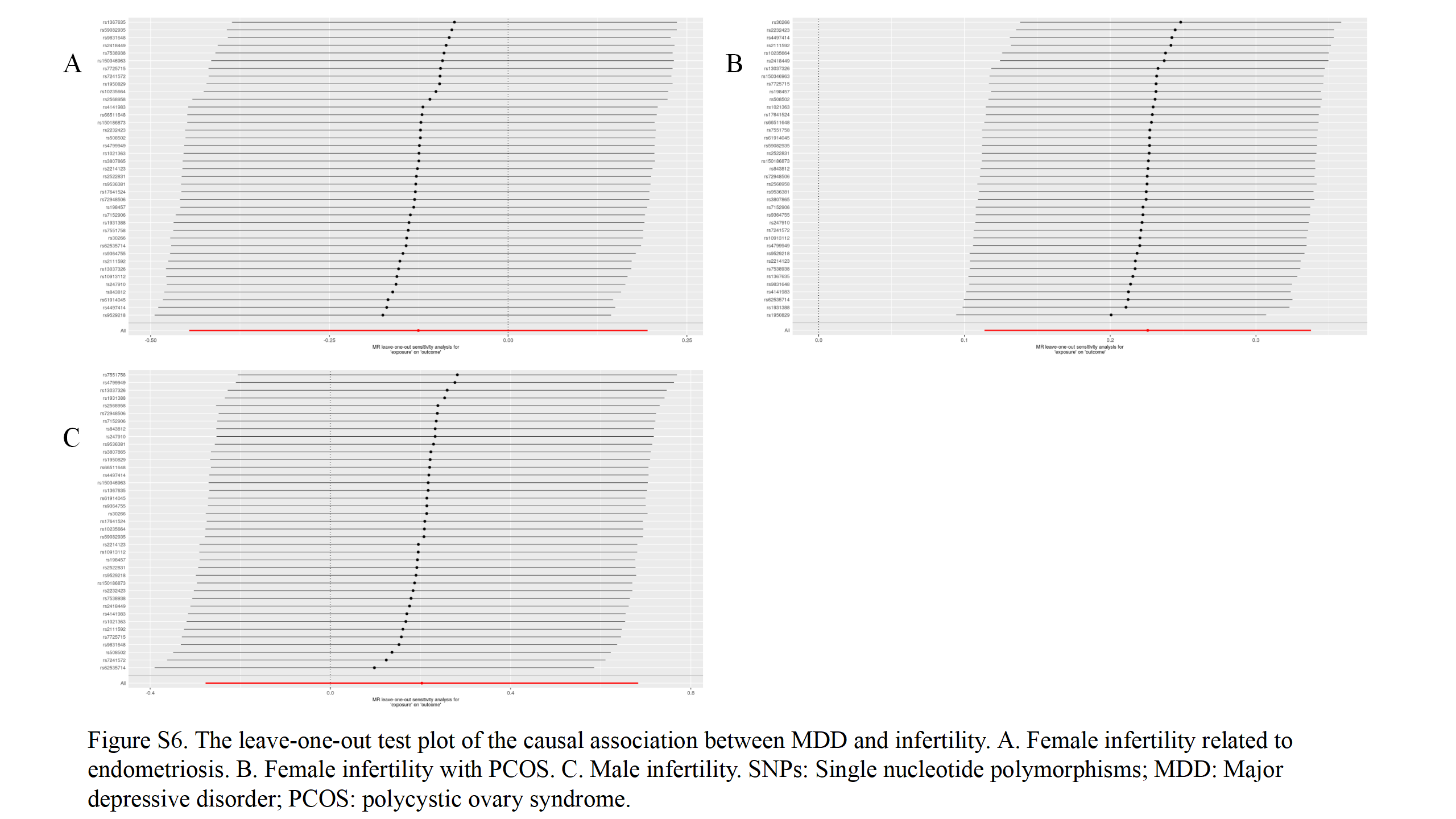

Supplement: Supplementary 12 — Figure 6: the funnel plot of SNPs associated with MDD and infertility: (a) female infertility related to endometriosis, (b) female infertility with PCOS, and (c) male infertility. SNPs, single nucleotide polymorphisms; MDD, major depressive disorder; PCOS, polycystic ovary syndrome. [file 9234876.f12.tif]

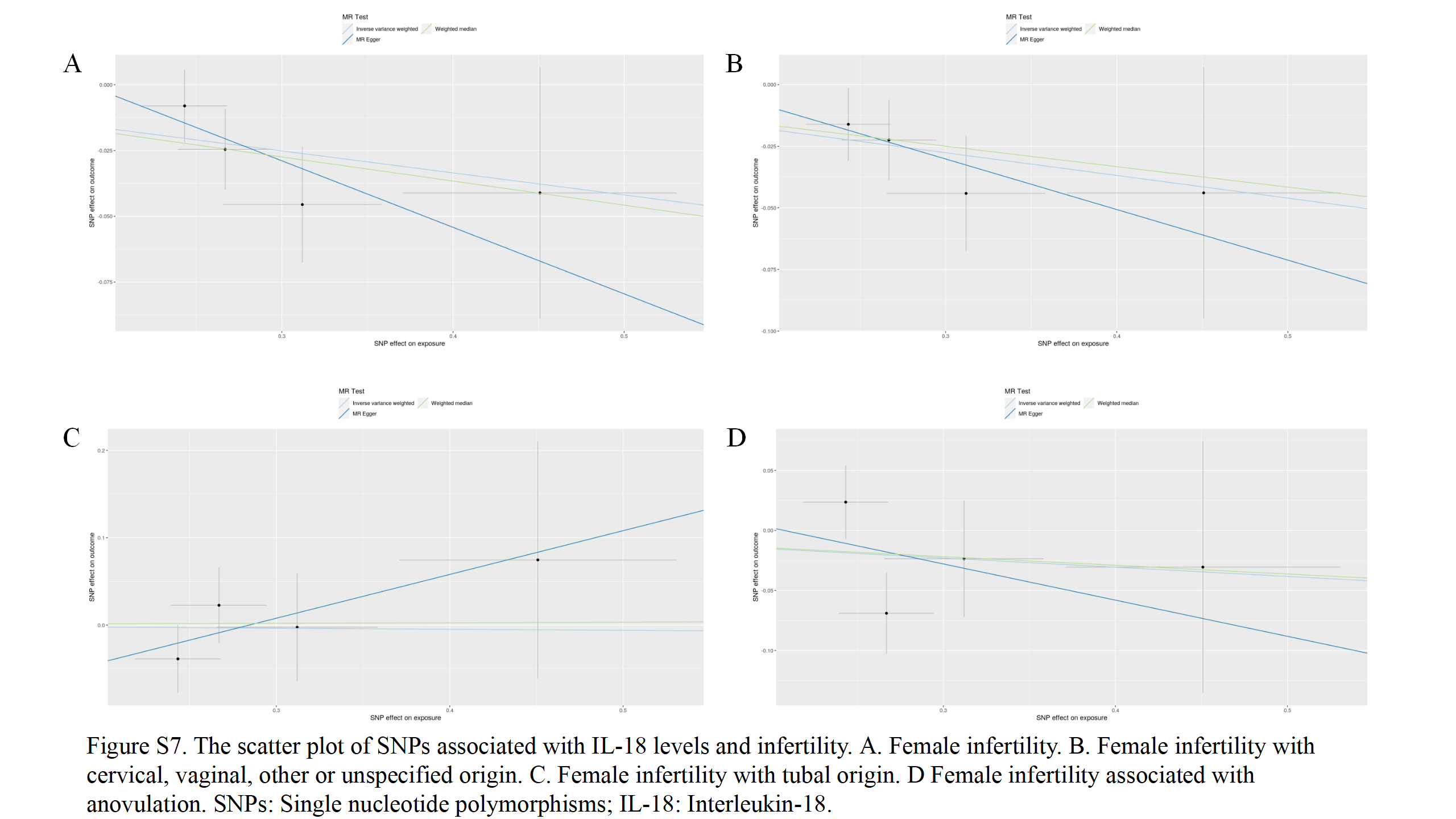

Supplement: Supplementary 13 — Figure 7: the funnel plot of SNPs associated with IL-18 levels and infertility: (a) female infertility, (b) female infertility with cervical, vaginal, other or unspecified origin, (c) female infertility with tubal origin, and (d) female infertility associated with anovulation. SNPs, single nucleotide polymorphisms; IL-18, interleukin-18. [file 9234876.f13.tif]

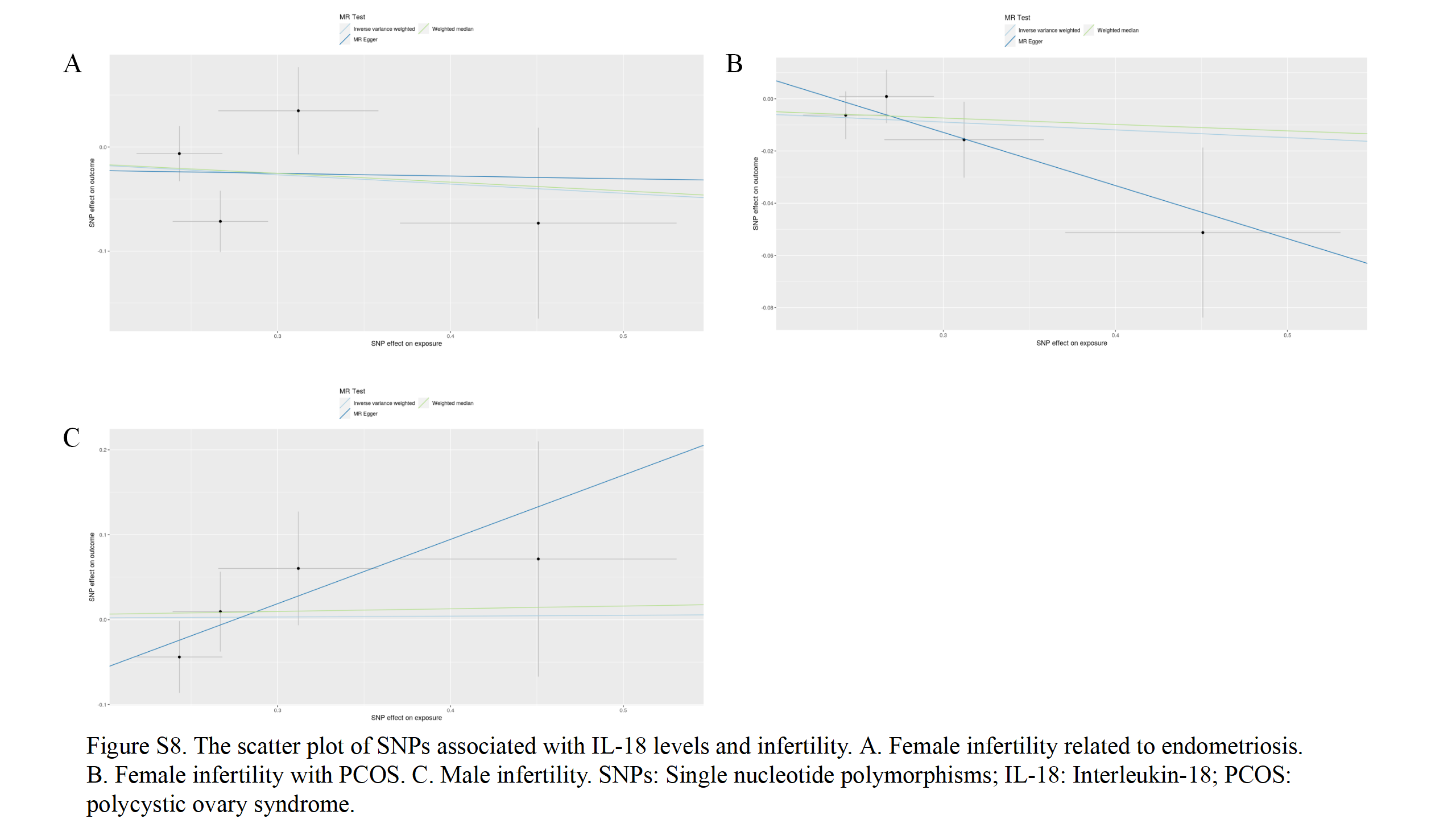

Supplement: Supplementary 14 — Figure 8: the funnel plot of SNPs associated with IL-18 levels and infertility: (a) female infertility related to endometriosis, (b) female infertility with PCOS, and (c) male infertility. SNPs, single nucleotide polymorphisms; IL-18, interleukin-18; PCOS, polycystic ovary syndrome. [file 9234876.f14.tif]

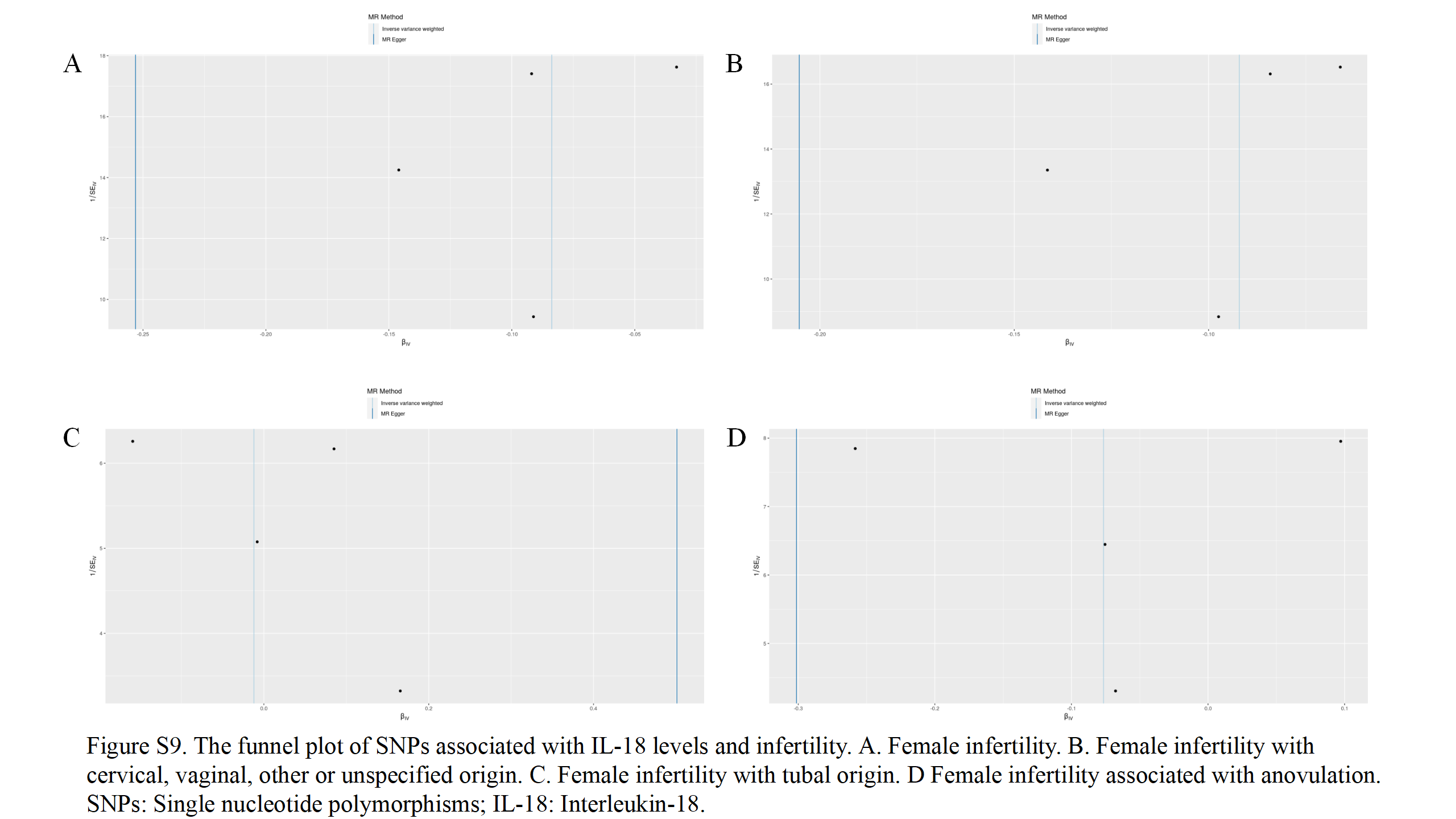

Supplement: Supplementary 15 — Figure 9: the leave-one-out test plot of the causal association between MDD and infertility: (a) female infertility, (b) female infertility with cervical, vaginal, other or unspecified origin, (c) female infertility with tubal origin, and (d) female infertility associated with anovulation. MDD, major depressive disorder. [file 9234876.f15.tif]

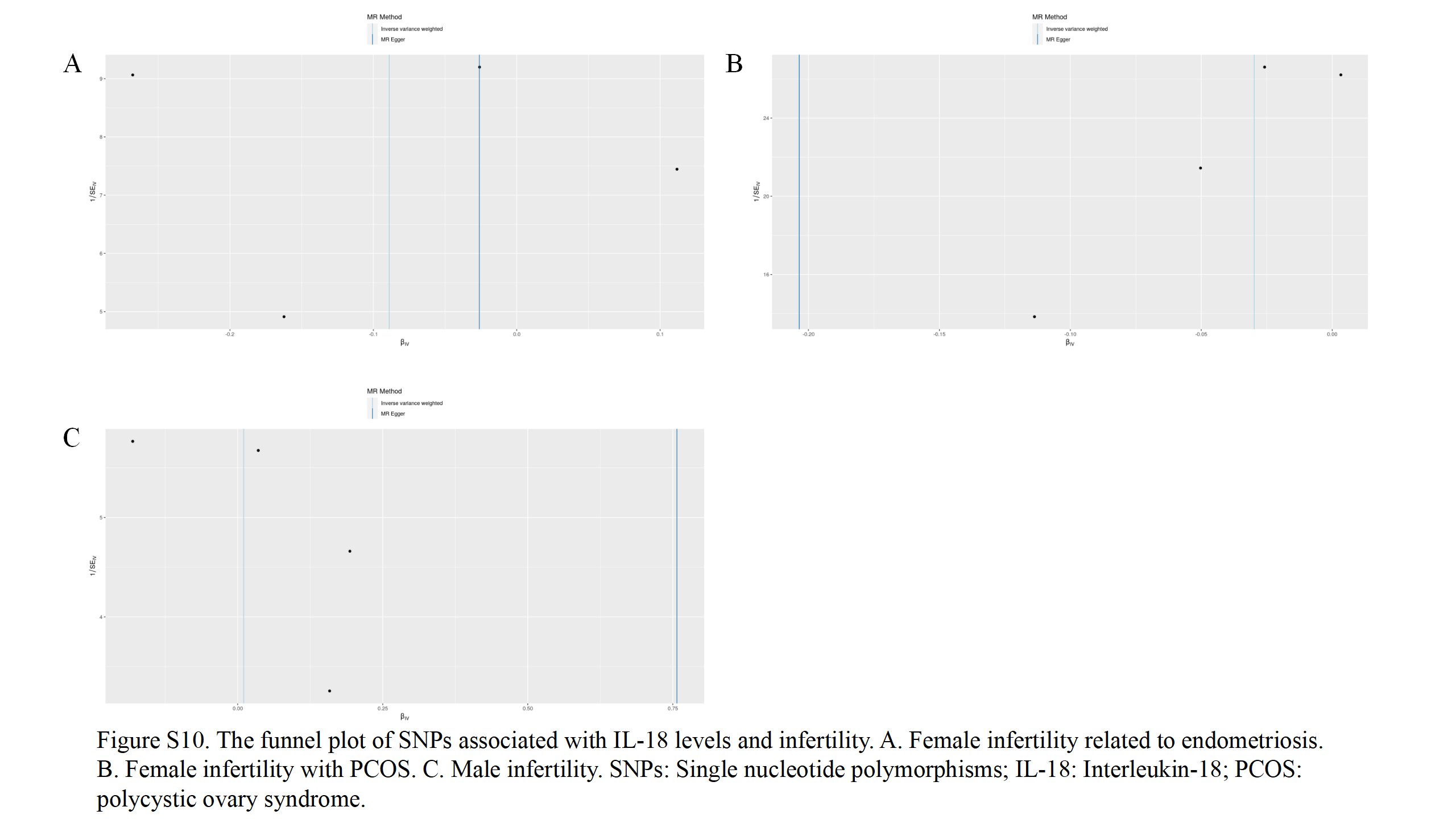

Supplement: Supplementary 16 — Figure 10: the leave-one-out test plot of the causal association between MDD and infertility: (a) female infertility related to endometriosis, (b) female infertility with PCOS, and (c) male infertility. SNPs, single nucleotide polymorphisms; MDD, major depressive disorder; PCOS, polycystic ovary syndrome. [file 9234876.f16.tif]

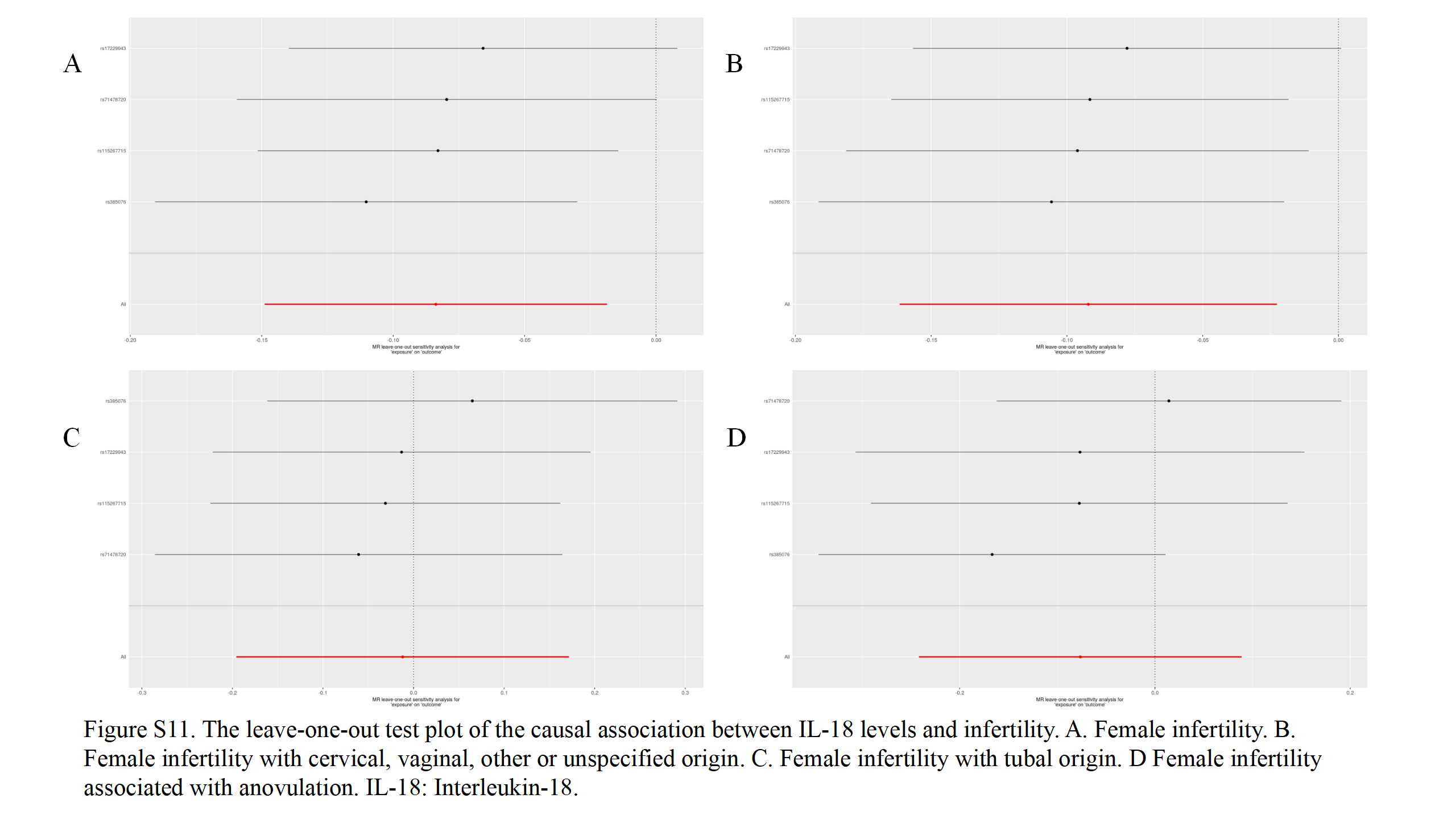

Supplement: Supplementary 17 — Figure 11: the leave-one-out test plot of the causal association between IL-18 levels and infertility: (a) female infertility, (b) female infertility with cervical, vaginal, other or unspecified origin, (c) female infertility with tubal origin, and (d) female infertility associated with anovulation. IL-18, interleukin-18. [file 9234876.f17.tif]

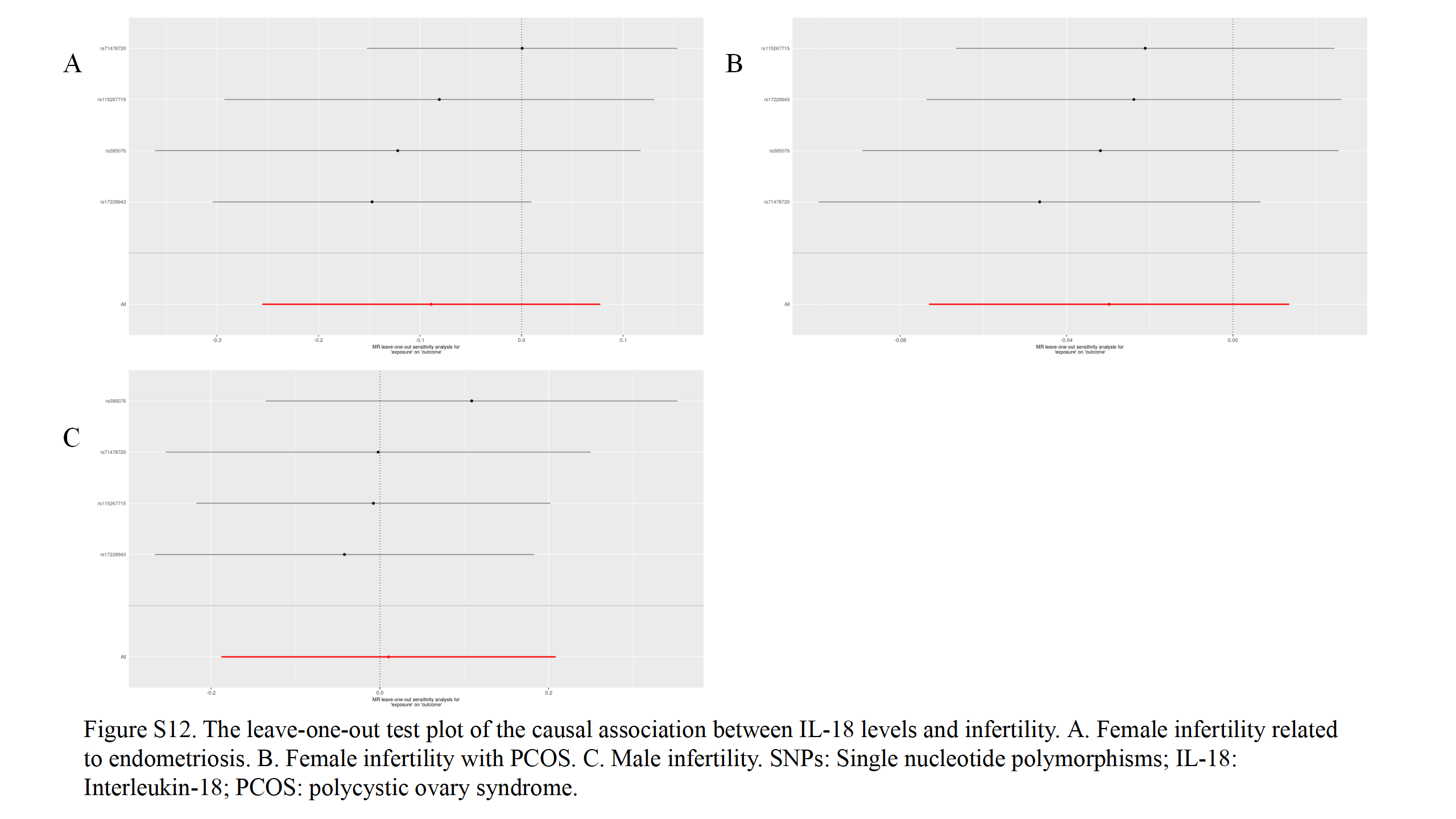

Supplement: Supplementary 18 — Figure 12: the leave-one-out test plot of the causal association between IL-18 levels and infertility: (a) female infertility related to endometriosis, (b) female infertility with PCOS, and (c) male infertility. SNPs, single nucleotide polymorphisms; IL-18, interleukin-18; PCOS, polycystic ovary syndrome. [file 9234876.f18.tif]
